# Enhanced enzymatic production of cholesteryl 6ʹ-acylglucoside impairs lysosomal degradation for the intracellular survival of *Helicobacter pylori*

**DOI:** 10.1186/s12929-021-00768-w

**Published:** 2021-10-27

**Authors:** Sasikala Muthusamy, Hau-Ming Jan, Ming-Yen Hsieh, Soumik Mondal, Wen-Chun Liu, Yi-An Ko, Wei-Yuan Yang, Kwok-Kong Tony Mong, Guang-Chao Chen, Chun-Hung Lin

**Affiliations:** 1 Institute of Biological Chemistry, Academia Sinica, No. 128 Academia Road Section 2, Nan-Kang, Taipei, 11529 Taiwan; 2 Molecular and Biological Agricultural Sciences Program, Taiwan International Graduate Program, National Chung-Hsing University and Academia Sinica, Taipei, 11529 Taiwan; 3 Graduate Institute of Biotechnology, National Chung-Hsing University, Taichung, 40227 Taiwan; 4 Biotechnology Center, National Chung-Hsing University, Taichung, 40227 Taiwan; 5 Institute of Biochemical Sciences, National Taiwan University, Taipei, 10617 Taiwan; 6 Department of Applied Chemistry, National Chiao Tung University, Hsin-Chu, 30010 Taiwan; 7 Department of Chemistry, National Taiwan University, Taipei, 10617 Taiwan; 8 Biomedical Translation Research Center, Academia Sinica, Taipei, 11529 Taiwan

**Keywords:** *H. pylori*, Cholesteryl glucosides, Autophagy, Autophagy flux, Lysosomes, Lysosome biogenesis, Autophagosomes, Lipid-raft clustering, Bacterial internalization

## Abstract

**Background:**

During autophagy defense against invading microbes, certain lipid types are indispensable for generating specialized membrane-bound organelles. The lipid composition of autophagosomes remains obscure, as does the issue of how specific lipids and lipid-associated enzymes participate in autophagosome formation and maturation. *Helicobacter pylori* is auxotrophic for cholesterol and converts cholesterol to cholesteryl glucoside derivatives, including cholesteryl 6ʹ-*O*-acyl-α-d-glucoside (CAG). We investigated how CAG and its biosynthetic acyltransferase assist *H. pylori* to escape host-cell autophagy.

**Methods:**

We applied a metabolite-tagging method to obtain fluorophore-containing cholesteryl glucosides that were utilized to understand their intracellular locations. *H. pylori* 26695 and a cholesteryl glucosyltransferase (CGT)-deletion mutant (ΔCGT) were used as the standard strain and the negative control that contains no cholesterol-derived metabolites, respectively. Bacterial internalization and several autophagy-related assays were conducted to unravel the possible mechanism that *H. pylori* develops to hijack the host-cell autophagy response. Subcellular fractions of *H. pylori-*infected AGS cells were obtained and measured for the acyltransferase activity.

**Results:**

The imaging studies of fluorophore-labeled cholesteryl glucosides pinpointed their intracellular localization in AGS cells. The result indicated that CAG enhances the internalization of *H. pylori* in AGS cells. Particularly, CAG, instead of CG and CPG, is able to augment the autophagy response induced by *H. pylori.* How CAG participates in the autophagy process is multifaceted. CAG was found to intervene in the degradation of autophagosomes and reduce lysosomal biogenesis, supporting the idea that intracellular *H. pylori* is harbored by autophago-lysosomes in favor of the bacterial survival. Furthermore, we performed the enzyme activity assay of subcellular fractions of *H. pylori*-infected AGS cells. The analysis showed that the acyltransferase is mainly distributed in autophago-lysosomal compartments.

**Conclusions:**

Our results support the idea that the acyltransferase is mainly distributed in the subcellular compartment consisting of autophagosomes, late endosomes, and lysosomes, in which the acidic environment is beneficial for the maximal acyltransferase activity. The resulting elevated level of CAG can facilitate bacterial internalization, interfere with the autophagy flux, and causes reduced lysosomal biogenesis.

**Supplementary Information:**

The online version contains supplementary material available at 10.1186/s12929-021-00768-w.

## Background

*Helicobacter pylori* is a human pathogen that infects more than half the population worldwide. Prolonged infection imposes a high risk for gastric cancer, which represents the second leading cause of cancer-related death worldwide [1]. Infection with *H. pylori* is also associated with other gastroduodenal diseases, including chronic gastritis, peptic ulcer, and mucosa-associated lymphoid tissue lymphoma [2–4]. Despite the pronounced defense mechanisms mounted by the human gastric tissue or immune system, the complete eradication of *H. pylori* generally is not achieved [5], which leads to bacterial colonization and persistence [1]. Sustained infection not only causes chronic inflammation but also creates a microenvironment predisposed to tumorigenesis, eventually resulting in gastric carcinoma. Although *H. pylori* persistence definitely plays a major role in overall pathogenesis, our understanding of the detailed mechanism remains incomplete.

*Helicobacter pylori* has long been considered as an extracellular pathogen, but research has demonstrated the existence of an intracellular population [6]. A considerable T helper 1 cell response—a signature of an invasive pathogen—is triggered by *H. pylori* infection [7]. Eradication of *H. pylori* becomes inefficient when infected cells in culture are treated with non-cell-permeable antibiotics [8]. Lipid rafts on the eukaryotic cell plasma membrane offer a safe portal for entry of many intracellular bacterial species, including *H. pylori* [9], *Porphyromonas gingivalis* [10]*, Mycobacterium tuberculosis* [11], and uropathogenic *Escherichia coli* FimH^+^ [12]. Lipid rafts are cholesterol-rich, nanoscale assemblies that affect plasma membrane features and functions, including curvature, transmembrane signaling, and endocytosis [13].

Autophagy acts as a cellular immune defense in response to diverse deleterious stimuli, resulting in the sequestration of intracellular pathogens in double-membrane, cytoplasm-containing autophagosomes [6, 14, 15]. The materials engulfed by phagosomes and/or autophagosomes are usually degraded by lysosomes [16]. Autophagosome formation is a dynamic, membrane-sculpting process in which lipids play an indispensable role. Notwithstanding that the lipid composition of autophagosomes is complex, certain lipids and lipid-associated enzymes are essential for the induction and expansion of autophagosome membrane and its ultimate fusion with lysosomes. For instance, the activation of cytosolic phospholipase A2 causes permeabilization of the lysosomal membrane in neurons after spinal-cord injury, and the damage to lysosomes results in the accumulation of neuronal autophagosomes, which contributes to neuronal cell death [17].

**Fig. 1:**
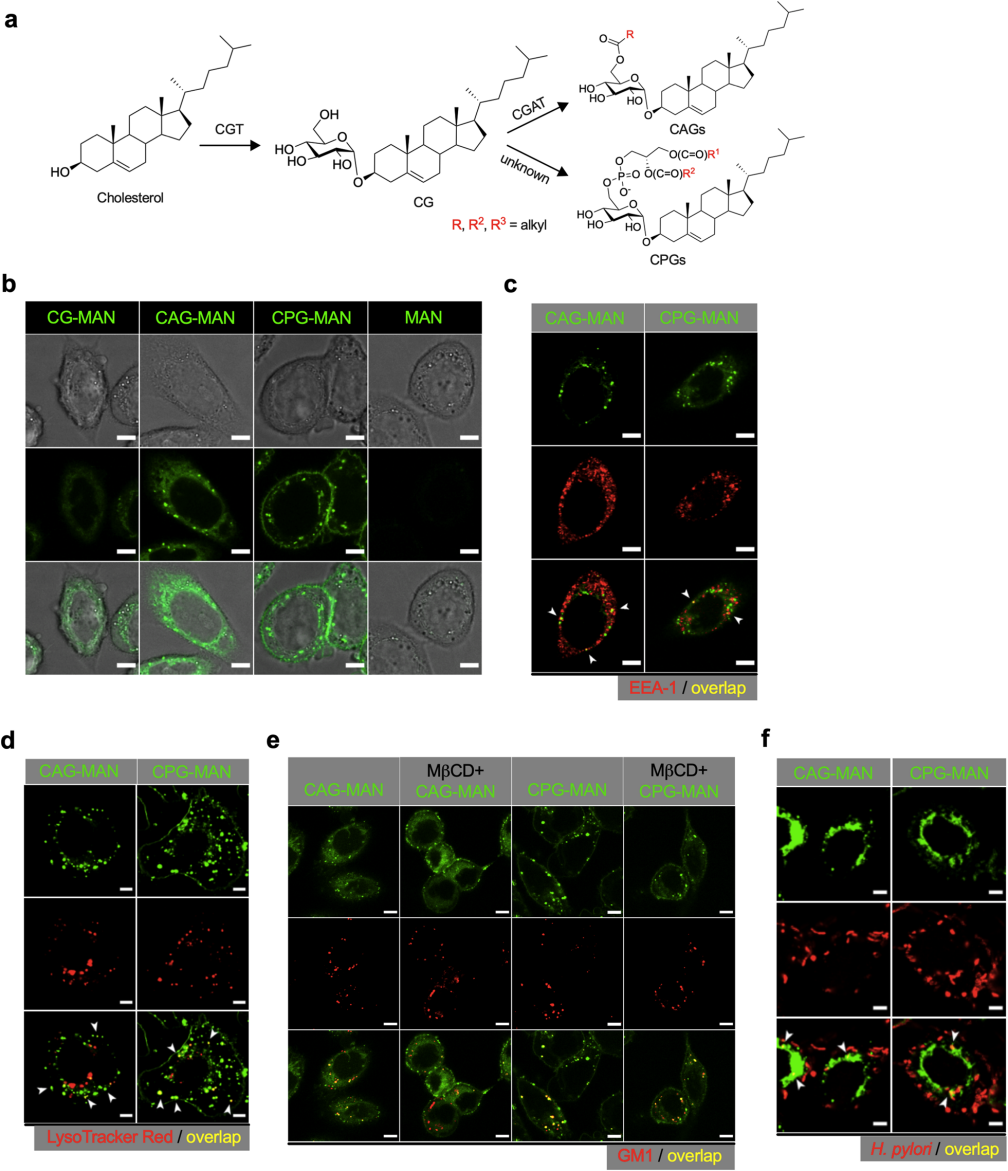
Subcellular localization of CAG and CPG in AGS cells. **a** Biosynthetic pathway for cholesterol α-glucoside derivatives in *H. pylori*. **b** Representative images of puncta in AGS cells. AGS cells were treated with CG-MAN, CAG-MAN, CPG-MAN, or MAN (green), fixed, and imaged with confocal microscopy. Scale bar: 5 μm. **c**, **d** CAG-MAN and CPG-MAN are internalized to early endosomes and subsequently routed to lysosomes. AGS cells were treated with CAG-MAN or CPG-MAN, followed by immunostaining for EEA1 (early endosome antigen 1, red) or Lysotracker Red™ (red) to detect early endosomes (**c**) and lysosomes (**d**), respectively. **e** Removal of membrane cholesterol abolishes CAG internalization. AGS cells were pretreated with (or without) methyl β-cyclodextrin (MβCD) to sequester membrane cholesterol for 1 h, stained with GM1 (red) for 30 min, washed three times, and treated with CAG-MAN (green) or CPG-MAN (green) for another 1 h. The cells were then fixed and imaged with confocal microscopy. Scale bar: 5 μm. **f** Overlapped images of *H. pylori* 26695 with the puncta of intracellular CAG-MAN or CPG-MAN. AGS cells were treated with CAG-MAN (green) or CPG-MAN (green) for 3 h and co-cultured with *H. pylori* 26695 for 2 h. The cells were then fixed and immunostained with anti-*H. pylori* (red). Arrowheads indicate co-localization of *H. pylori* with puncta. Scale bar: 5 μm

*Helicobacter pylori* is a cholesterol auxotroph and thus must assimilate cholesterol from the membranes of host epithelial cells [18]. *Helicobacter* species have evolved along with a unique biosynthetic pathway for cholesterol modifications, in which cholesteryl glycosyltransferase (CGT; *hp0421* is the corresponding gene) catalyzes the glucosylation of cholesterol to form cholesteryl α-d-glucopyranoside (CG) [19, 20]. An acyl or phosphatidyl group is further attached to the 6ʹ-OH of glucose, leading to the formation of cholesteryl 6ʹ-*O*-acyl-α-d-glucopyranoside (CAG) or cholesteryl 6ʹ-*O*-phosphatidyl-α-d-glucopyranoside (CPG), respectively. The enzyme responsible for the formation of CAG is cholesteryl α-d-glucopyranoside 6′-acyltransferase (CGAT), whereas the enzyme for CPG formation remains unknown (Fig. 1a). CGAT is a bifunctional enzyme that catalyzes the hydrolysis of phospholipids and the subsequent transfer of the acyl group [21]. We previously developed a metabolite-tagging method to identify a variety of cholesteryl glucoside derivatives (CGds) with *femto*molar sensitivity. We found that *H. pylori* infection of epithelial cells remarkably alters CAG composition [22]. This change occurs in concert with enhancement of lipid-raft clustering on the host-cell plasma membrane, thus gathering adhesion molecules and in turn favoring *H. pylori* adhesion [21].

We herein report evidence that CGAT was internalized and mainly distributed in a subcellular compartment in gastric epithelial cells. Because CGAT activity is optimal at low pH, the enzyme appears to enhance the production of CAG in autophagosomes and lysosomes. Additionally, CAG contributes to *H. pylori* internalization, disrupts autophagy flux, and interferes with the degradation in lysosomes, which together favor the survival of the intracellular bacterium. These findings pinpoint CGAT as a potential target for therapeutic intervention.

## Materials and methods

### Bacterial culture, cell culture, and co-culture

*Helicobacter pylori* cells were regularly cultured on CDC Anaerobic Blood Agar plates (Becton Dickinson, New Jersey, USA) under microaerobic (Anaeropack Campylo System) and humidified conditions at 37 °C for 2 days. *H. pylori* 26695 (*ATCC*^*®*^ 700392™) and ΔCGT (*hp0421*-knockout) were used in this study. ΔCGT was generated as described previously [21, 23]. To analyze the bacterial growth, each strain of bacteria (3 × 10^7^) was incubated in 2 ml of *Brucella* broth supplemented with 10% fetal bovine serum (FBS) at 37 °C, 200 rotation per minute (RPM) under microaerophilic condition. Samples collected at 0, 3, 8, 12, 28 h were serially diluted and plated on CDC Anaerobic Blood Agar plates. Number of viable colonies were counted after 5 days and shown as CFU/ml.

AGS cells (purchased from American Type Culture Collection (ATCC, Virginia, USA) (ATCC^®^ CRL-1739™)) and GES-1 cells (a kind gift by Dr. Deng-Chiang Wu at Kaohsiung Medical University Hospital) were maintained in a humidified environment with 5% CO_2_ at 37 °C. The cells were cultured in Dulbecco’s Modified Eagle Medium (DMEM, Gibco, California, USA) supplemented with 10% endotoxin-free fetal bovine serum (HyClone, Logan, USA) and a 1% mixture of penicillin–streptomycin–ampicillin (Biological Industries, Israel). Cells tested negative for mycoplasma by PCR. For co-culture experiments, we utilized the medium that did not contain the aforementioned antibiotics and serum [22].

### Antibodies and reagents

Antibodies used in this work are commercially available, as follows: from Abcam (Cambridge, UK), anti-*H. pylori* (ab20459) [24], rabbit anti-Lamp-1 (ab25030-100), mouse anti-EEA1 (ab2900), rabbit anti-Rab7 (ab50533), rabbit anti-Na^+^K^+^ ATPase (ab76020), rabbit anti-cathepsin D (ab75852), and rabbit anti-histone H3 (ab1791); from Sigma-Aldrich (Missouri, USA), rabbit anti-LC3B (L7543). Additionally, the following reagents were purchased: LysoTracker^®^ Red DND-99 (Invitrogen, California, USA; L7528), cholera toxin B staining kit (Invitrogen; V34404), Magic Red™ cathepsin B staining kit (Bio-Rad, California, USA; ICT937), Oregon Green™ 488 BAPTA-5N (Invitrogen California, USA; O6812), Dextran Alexa Fluor™ 568 (Invitrogen California, USA; D22912), LysoSensor™ Yellow/Blue DND-160 (Invitrogen California, USA; L7545), 3-methyl adenine (Sigma; M9281), chloroquine (Sigma; C6628), rapamycin (Sigma; R8781), bafilomycin-A1 (Sigma; B1793), and concanamycin A (Sigma; C9705).

### Preparation and purification of CG-MAN, CAG-MAN, and CPG-MAN from *H. pylori*

The purification was carried out as described elsewhere [22]. Briefly, *H. pylori* cells were cultured for 2–4 days on blood agar plates containing 17β-([3″-azidopropoxy)-5-androsten-3β-ol (an azide-containing cholesterol analog, 50 μM). Bacterial lipids were extracted using the Folch method [22], redissolved in chloroform/methanol/water (5:4:1, v/v/v), and subjected to a click reaction with an alkyne-containing dye (4-*N*-methylamino-1,8-napthalimidopropyne, 0.25 mM), and chromatographed with preparative HPLC to afford CG-MAN, CAG-MAN, and CPG-MAN according to an established procedure [22].

### Pretreatment of cells with CGds and subsequent bacterial infection

Authentic CG and CAG, and CPG (CPG-MAN) were synthesized in accordance with the reported procedure [22]. Cells were seeded on culture plates containing Dulbecco’s modified Eagle’s medium (Gibco, Invitrogen) and cultured for 16 h at 37 °C. Cells were treated with CAG (or any of the CGds, 20 μM) in serum-free Ham’s F-12 nutrient medium (F-12 medium) or DMEM for 1 h and co-cultured with *H. pylori* (MOI: 100) in F-12 medium or DMEM for an additional 6 h. The cells were then washed several times with Dulbecco’s phosphate-buffered saline (DPBS) and treated with gentamycin (200 μg/ml) for 1 h to kill the adhered *H. pylori*. After washing with DPBS, the cells were collected for further studies. When cells were treated with CAG, CAG (16:0) that contain a palmitic acyl chain was used unless otherwise mentioned.

### Immunofluorescence microscopy

AGS cells or GES-1 cells (2 × 10^5^) were seeded on cover glasses in a 12-well format and incubated for 16 h in a CO_2_ incubator. For staining of lipid rafts, AGS cells were treated with methyl-β-cyclodextrin (5 mM) for 1 h, stained with cholera toxin B (conjugated with Alexa Fluor 594 (red)) at 4 °C for 30 min, and incubated with CAG-MAN (green) or CPG-MAN (green) for 1 h at 37 °C. Clustering of lipid rafts, shown by the staining of GM1, was visualized with a Leica SP5 X confocal microscope. To label lysosomes, AGS cells were pretreated with CAG or any of the CGds, infected with *H. pylori*, and then stained with LysoTracker™ Red DND-99 for 30 min. The cells were then imaged under a Leica SP5 X confocal microscope, and the signals were quantified using Image J software. Alternatively, AGS cells or GES-1 cells were pre-treated with DMSO or CAG, followed by infection with *H. pylori*. The fixed cells were then immunostained for Lamp-1 and imaged with confocal microscopy. To examine autophagosomes and lysosomes, AGS cells were transiently transfected with EGFP-tagged LC3 plasmid or RFP-tagged TPC2 plasmid for 16 h with Lipofectamine™ 2000 (Invitrogen), treated with CAG, and infected with *H. pylori*. Confocal microscopy (Leica SP5 X) was then used to examine puncta structures. To monitor the activity of the lysosomal protease cathepsin B, AGS cells were pretreated with CAG for 1 h, infected with *H. pylori* for 6 h, treated with Magic Red™ Cathepsin B substrate (red), and imaged immediately with confocal microscopy. For all immunofluorescence staining, AGS cells were fixed with 2% formaldehyde, permeabilized with 0.1% (v/v) Triton-X 100 or 0.1% saponin, and labeled with anti-Lamp-1 (1:500) or anti-EEA1 (1:200) at 4 °C for 16 h. After incubation with a specific secondary antibody tagged with Alexa Fluor 488 (green) or Alexa Fluor 647 (red), the cells were imaged with confocal microscopy. Image J software was used for quantification.

### Autophagy flux assay

To assay autophagy flux, AGS cells (2 × 10^5^) were seeded and incubated for 16 h, transfected with mRFP-GFP-LC3 plasmid for an additional 16 h, and treated with CAG. The cells were then infected with *H. pylori* for 6 h, fixed, and examined under a confocal microscope. Images were quantified with Image J software. The signals of yellow and red were defined as follows. Yellow signals came from the puncta count of GFP^+^RFP^+^ (autophagosomes), whereas red signals resulted from the puncta count of RFP^+^ (autolysosomes). Autophagy flux was indexed by the yellow/red ratio.

### Bacterial internalization assay and gentamycin colony formation unit assay

AGS cells (6 × 10^5^) were seeded on 6-well plates and cultured for 16 h. Total bacteria that included well-adhered and intracellular bacteria were counted as follows. AGS cells were pretreated with CAG (or any of the CGds) for 1 h and infected with *H. pylori* (MOI: 100) in F-12 medium for 2, 4 or 6 h. The cells were then washed five times with DPBS, lysed with 0.1% saponin, and plated on blood agar plate to estimate the number of total bacteria as CFU/ml. Intracellular bacteria were counted as follows. After pretreatment with CAG (or any of the CGds), AGS cells were infected with *H. pylori* for 2, 4 or 6 h, washed five times with DPBS, and cultured in the presence of gentamycin (200 μg/ml) for 1 h to kill extracellular bacteria. The cells were then lysed with 0.1% saponin and plated on blood agar plates to estimate the number of intracellular bacteria as CFU/ml.

### Immunoblotting

AGS cells (6 × 10^5^) or GES-1 cells (4 × 10^5^) were seeded on 6-well plates for 16 h and infected with *H. pylori* (MOI 100) for 6 h. The infected cells were washed five times with DPBS, and whole-cell lysates were prepared in radioimmunoprecipitation assay buffer (Sigma-Aldrich) with protease inhibitor cocktail (Calbiochem) and phosphatase inhibitor cocktail (Calbiochem). The samples were then analyzed by SDS-PAGE. The proteins were then transferred to a polyvinylidene difluoride membrane (GE Healthcare). Each membrane was blocked with 5% (w/v) dry milk in Tris-buffered saline (50 mM Tris, 150 mM NaCl, 1 mM CaCl_2_, pH 7.4) containing 0.01% (v/v) Tween-20 at room temperature for 1 h and incubated at 4 °C for 16 h with rabbit monoclonal anti-LC3B (Sigma; 1:4000), mouse anti-β-actin (Sigma; 1:5000), rabbit anti-Cathepsin D (Abcam; 1:5000), rabbit anti-Lamp-1 (Abcam; 1:1000) or rabbit anti-GAPDH (Abcam; 1:5000). Each blot was washed three times and incubated at room temperature for 1 h with the appropriate horseradish peroxidase-conjugated secondary antibody (Santa Cruz Biotechnology; 1:5000). The blots were visualized with ECL western blotting detection reagents (Millipore; WBKLS0500) and visualized with a Luminescent Image Analyzer (LAS4000, Fujifilm).

### Subcellular fractionation and measurement of CGAT activity

AGS cells (2 × 10^7^) co-cultured with *H. pylori* 26695 were lysed and fractionated with the Minute™ Plasma Membrane Extraction and Subcellular Fractionation kit (Invent Biotechnologies, Minnesota, USA). Four fractions were obtained from the whole-cell lysate, including nucleus, cytosol, organelles, and plasma membrane. Proteins were quantified with the BCA assay. An aliquot of each fraction (20 µg) was analyzed by immunoblotting. The quality of the subcellular fractions was assessed using specific marker antibodies. CGAT activity in whole-cell lysate and each subcellular fraction was measured according to a previous report [21] and presented as specific activity (fmol/min/μg). The distribution of CGAT activity was measured by calculating the ratio of activity in each fraction to the total activity (sum of the activities of all subcellular fractions activity).

### Measurement of intra-luminal lysosomal Ca^2+^ levels

Intra-luminal lysosomal calcium levels were measured by Oregon Green 488 BAPTA 5 N and 10 kDa Dextran-conjugated with Alexa Fluor-586 [25]. In 96-well cell culture plate, GES-1 cells (3 × 10^4^) were added with the membrane-impermeant Oregon Green BAPTA-5N (Ca^2+^ indicator probe; 15 μM) and Alexa Fluor-586-conjugated dextran (pH insensitive endocytic probe; 0.25 mg/ml) for 2 h. The cells were then treated with DMSO or CAG for 1 h, infected with *H. pylori* 26695 or ΔCGT for 6 h. Cells treated with bafilomycin A1 (20 nM) and concanamycin A (10 nM) were used as positive controls. The Oregon Green signal was recorded by fluorescence with the excitation at 488 nm and emission at 526 nm. To capture the fluorescent signals of Dextran-conjugated with Alexa Fluor-586, the excitation and emission wavelengths were set at 568 and 603 nm, respectively. The relative calcium levels were shown as a ratio of Oregon Green/ Dextran Red.

### Measurement of pH levels of lysosomes

Lysosomal pH was measured using Yellow/Blue DND-160 [26]. In 96-well plate, GES-1 cells (3 × 10^4^) were loaded with 10 μM of the probe for 1 h, treated with CAG or DMSO, and infected with *H. pylori* 26695 or ΔCGT for 6 h. Cells treated with bafilomycin A1 (20 nM) and concanamycin A (10 nM) were used as positive controls. The signals were measured by fluorescence with the excitation at 329 nm and the dual emissions recorded at 440 and 540 nm. The relative LysoSensor™ Yellow/Blue ratio was calculated as the ratio of the two emission signals.

### Mouse infection to measure *H. pylori* colonization

Six-week-old, specific-pathogen-free, and *Helicobacter*-free C57BL/6 male mice were obtained from BioLASCO Taiwan Co., Ltd. and housed at Infectious Disease Core Facility in Biomedical Translation Research Center in Taiwan. *H. pylori* 26695 and ΔCGT (10^10^ bacteria in 200 μl of *Brucella* broth) were administered by intragastrically for 3 consecutive days for 2 weeks [27, 28]. Mice were euthanized 10 weeks post-the infection. The stomachs were halved longitudinally, and rinsed in sterile DPBS. Half of the stomach was homogenized using a mechanical homogenizer in *Brucella* broth containing 10% FBS at room temperature. The appropriate volume of serial dilutions was plated on *Brucella* agar supplemented with 10% FBS, *H. pylori*-selective supplement (Dent Oxoid™; SR0147), and 2.5 international unit (IU) ml^−1^ polymyxin B to determine colonization (CFU) [26]. The resulting values were normalized by the weight of the fresh tissue used (CFU/mg). The other half of the stomach was formalin-fixed, embedded in optimum cutting temperature compound (OCT), and sectioned to perform immunofluorescence for *H. pylori* and the autophagy marker LC3.

### Statistical analysis

Statistical significance between two samples was tested by an unpaired t test. Prism 8 software (GraphPad, La Jolla, CA) was used for statistical analysis. All statistically significant differences are indicated with asterisks: *p < 0.05, **p < 0.01; *p* > 0.05, not significant.

## Results

### Imaging studies of fluorophore-labeled cholesteryl α-glucosides pinpoint their intracellular localization in AGS cells

CGds constitute a substantial portion of membrane lipids in *H. pylori*. Because CGAT catalyzes the reaction to produce CAG, we first examined the effect of CAG, and compared CAG with other CGds. To perform imaging studies of host AGS cells (human gastric cancer line), we applied a previously developed method to generate and purify the highly fluorescent analogs of CG, CAG, and CPG, which contain 4-*N*-methylamino-1,8-napthalimidopropyne (MAN) at the steroid moiety, to serve as the fluorophore [22]. Hereafter, these compounds are referred to as CG-MAN, CAG-MAN, and CPG-MAN, respectively (Additional file 1: Fig. S1).

We treated AGS cells with CG-MAN, CAG-MAN, or CPG-MAN (20 μM) for 1 h and monitored the subcellular localization of each metabolite by confocal microscopy. Interestingly, CAG-MAN and CPG-MAN had distinct patterns of intracellular puncta. CPG-MAN was also seen on the plasma membrane (Fig. 1b). Staining for the early endosome marker ‘early endosome antigen 1’ (EEA-1) (Fig. 1c) and Lysotracker Red™ (Fig. 1d) suggested that CAG-MAN and CPG-MAN were internalized through early endosomes and eventually delivered to lysosomes. CAG has been reported to promote the clustering of lipid rafts on the plasma membrane [22]. To understand if this documented internalization is relevant to lipid rafts, AGS cells were depleted of cholesterol using methyl β-cyclodextrin (MβCD, a cholesterol-sequestering agent), stained for lipid rafts with cholera toxin B subunit, and treated with CAG-MAN or CPG-MAN. Confocal microscopy revealed the co-localization of puncta substructures with lipid rafts (Fig. 1e) in the absence of MβCD, suggesting that both CAG-MAN and CPG-MAN were internalized via a lipid raft-dependent mechanism. Upon treatment with MβCD, CAG-MAN was mainly found on the plasma membrane, supporting the idea that cholesterol and clustering of the cholesterol-rich lipid rafts are essential for CAG internalization. On the other hand, the puncta pattern of CPG-MAN was essentially unaltered by MβCD. To confirm this result, AGS cells were treated with U18666A (inhibitor of cholesterol biosynthesis) before the addition of CAG-MAN or CPG-MAN, which led to the disappearance of puncta substructures of both CAG-MAN and CPG-MAN (Additional file 1: Fig. S2). These observations suggested that CPG-MAN also relies on host cholesterol for its uptake. The seemingly contradictory observations for CPG-MAN are likely attributable to the quick renewal of cellular cholesterol after the withdrawal of MβCD. Nevertheless, cholesterol homeostasis appeared to be disrupted upon U18666A treatment owing to its inhibitory effect on cholesterol biosynthesis and transport. Collectively, these studies demonstrated the importance of cholesterol and the clustering of lipid rafts for the cellular uptake of CAG-MAN and CPG-MAN. Next, AGS cells were treated with CAG-MAN or CPG-MAN, infected with *H. pylori* 26695, and imaged with confocal microscopy. The puncta of each of CAG-MAN and CPG-MAN co-localized with the intracellular bacteria, which were immunostained with an antibody against *H. pylori* (Fig. 1f and Additional file 1: Fig. S3a). Therefore, it is likely that CAG-MAN or CPG-MAN (or both) is necessary for bacterial internalization.

### CAG enhances the internalization of *H. pylori* in AGS cells

**Fig. 2:**
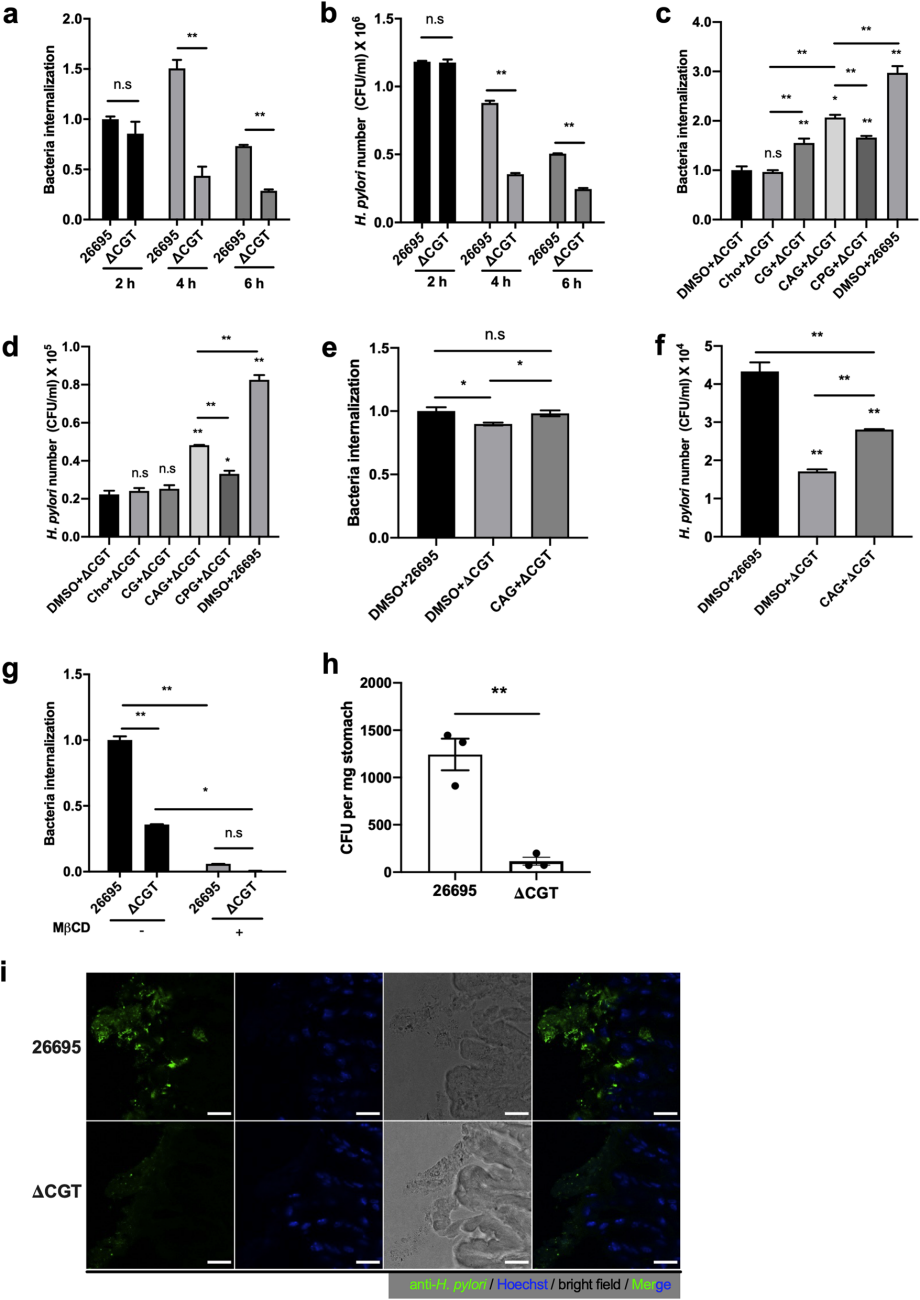
Treatment of AGS cells with CAG increases the number of intracellular *H. pylori*. **a** Bacterial internalization is reduced in the absence of cholesterol glucosides. AGS cells were co-cultured with *H. pylori* 26695 or ΔCGT for 2, 4 or 6 h. The samples were then lysed at the indicated time points and plated on blood agar plates for counting viable colonies. Bacterial internalization is presented as the proportion of intracellular bacteria out of the total bacteria.** b** To study the effect on the number of intracellular bacteria, AGS cells were co-cultured with *H. pylori* 26695 or ΔCGT for 2, 4 or 6 h and then treated with gentamycin for 1 h. Samples were lysed, and lysates were plated on blood agar plates for colony counting to give the number of intracellular bacteria (CFU/ml). **c** Bacterial internalization depends on CAG. AGS cells were first treated with cholesterol, CG, CAG, or CPG for 1 h, and then infected with *H. pylori* 26695 or ΔCGT for an additional 3 h. Bacterial internalization was then measured as mentioned in (**a**). **d** To measure the number of intracellular bacteria, AGS cells were first treated with CG, CAG, or CPG and co-cultured with *H. pylori* 26695 or ΔCGT for 6 h, followed by gentamycin treatment. The number of intracellular bacteria was then measured as mentioned in (**b**). **e** GES-1 cells were treated with DMSO or CAG, and then infected with *H. pylori* 26695 or ΔCGT. The degree of bacterial internalization was measured as described in (**a**). **f** GES-1 cells were treated with DMSO or CAG, infected with *H. pylori* 26695 or ΔCGT, and then subjected to the measurement for the number of intracellular bacteria as described in (**b**). **g** Bacterial internalization was dependent on lipid rafts. After AGS cells were treated with MβCD and infected with *H. pylori* 26695 or ΔCGT for another 3 h, internalized bacteria were counted. The data shown in panels (**a**–**c**, **e** and **g**) are normalized relative values. **h** ΔCGT greatly reduced *H. pylori* colonization in the mice stomach. Six-week-old C57BL/6J mice were intragastrically infected with *H. pylori* 26695 or ΔCGT. At 10 weeks post-the infection, the stomach samples were processed for CFU enumeration as described in “Materials and methods”. The number of viable colonies were counted and shown as CFU/mg of mice stomach (n = 3). Data shown represent the mean ± SEM (standard error of the mean). **p < 0.01, *p < 0.05 vs. the control (n = 3); n.s. (not significant). **i** The cryosections of *H. pylori* 26695- and ΔCGT-infected mice stomach samples were stained with anti-*H. pylori* antibody (green) and for nucleus (blue), and imaged by confocal microscopy. Scale bar: 20 μm

The internalization of *H. pylori* has been associated with its pathogenesis, including inflammation, colonization, and overall disease outcome [29]. A CGT-deletion mutant (ΔCGT) that does not produce any CGds was reported to have impaired adhesion [21, 23] to epithelial cells and invasion [30] of macrophages. We thus examined whether and how CGds are involved in bacterial internalization. Bacterial internalization was estimated as the ratio of the intracellular bacterial count (CFU assay in the presence of the cell-impermeable antibiotic gentamycin, to eradicate extracellular bacteria) [26, 30–32] to total bacterial count (CFU assay without gentamycin) [30, 33]. AGS cells were infected with *H. pylori* 26695 or ΔCGT, and at 4 h and 6 h post-infection the ΔCGT-infected cells displayed a significantly reduced level of internalization (Fig. 2a) and fewer intracellular bacteria (Fig. 2b) in comparison with those infected with *H. pylori* 26695. To identify which of the CGds played a role in internalization, AGS cells were treated with CG, CAG, or CPG for 1 h, infected with *H. pylori* 26695 or ΔCGT, and assayed for internalization (Fig. 2c) and for the number of intracellular bacteria (Fig. 2d). The result indicated that CAG, rather than CG or CPG, was critical for enhancing bacterial internalization (Fig. 2c) and increasing the number of intracellular *H. pylori* (Fig. 2d). GES-1 cells were utilized as non-cancerous epithelial cells for the same purpose. The cells were treated with CAG and infected with *H. pylori* in the same way. In consistence with the aforementioned result from AGS cells, the presence of CAG (i.e., *H. pylori* 26695 and CAG + ΔCGT) exhibited a higher level of bacterial internalization (Fig. 2e) and more intracellular bacteria (Fig. 2f). Because the observed difference might be caused by a possible growth defect of the knockout mutant, we thus examined the growth pattern of *H. pylori* 26695 and ΔCGT in *Brucella* broth supplemented with 10% FBS. The bacterial samples were collected at 0, 3, 8, 12 and 24 h, and then plated to count the number of viable *H. pylori* colonies. The result indicated no difference in their growth pattern at the used settings (Additional file 1: Fig. S3b), which was consistent with previous study that ΔCGT displayed no growth defect despite an altered morphology [34]. Therefore, the possibility of growth defect was excluded.

We previously reported that CAG promotes the clustering of lipid rafts on the host-cell plasma membrane, with subsequent recruitment of adhesion molecules (including integrins α5 and β1, and Lewis antigens) to enhance *H. pylori* adhesion [21]. Because bacterial adhesion is a prerequisite for internalization and cholesterol is essential for clustering of lipid rafts, we examined how cholesterol-rich lipid rafts affected bacterial internalization. AGS cells were treated with MβCD and then assayed for the degree of internalization. The MβCD-treated cells indeed exhibited lesser bacterial internalization than the untreated cells (Fig. 2g), which is consistent with previous reports [30, 35]. Collectively, these results suggested that *H. pylori* is internalized through a lipid raft-mediated mechanism.

To understand the in-vivo relevance and the importance of CGds for the *H. pylori* colonization of the stomach, C57BL/6 mice was utilized as an animal model. Each group of mice were intragastrically infected with *H. pylori* 26695 or ΔCGT, as described in “Materials and methods” [27, 28]. Ten weeks post-the infection, stomach samples were homogenized in *Brucella* broth supplemented with 10% FBS and plated to enumerate the number of viable *H. pylori* colonies (Fig. 2h). Next, the formalin-fixed cryo-sections of *H. pylori*-infected stomach samples were immunostained for *H. pylori* and imaged with confocal microscopy (Fig. 2i). Taken together, these results indicated that colonization of ΔCGT was significantly lesser than that of *H. pylori* 26695. In agreement with previously reported data [18], CGT or the corresponding CGds appear to be an important factor for *H. pylori* colonization of the mice stomach.

### CAG, instead of CG and CPG, enhances the autophagy response induced by *H. pylori*

**Fig. 3:**
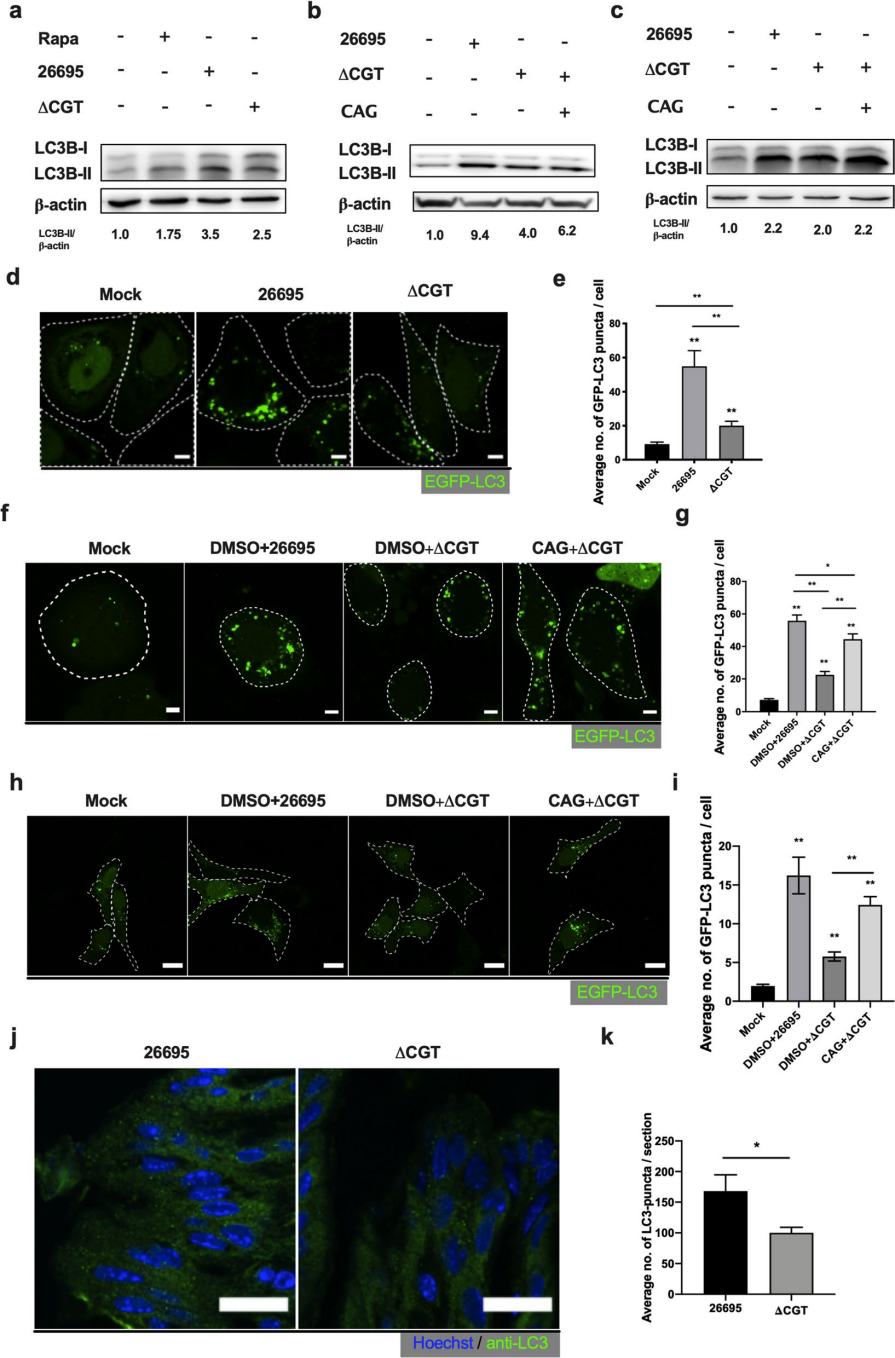
CAG enhances *H. pylori–*induced autophagy. **a** AGS cells were infected with *H. pylori* 26695 or ΔCGT (MOI: 100) for 6 h. The protein levels of LC3B-II and β-actin were measured by immunoblotting. Cells treated with rapamycin (100 nM) were used as the positive control. **b** AGS cells were treated either with DMSO or CAG for 1 h and infected with *H. pylori* as described in (**a**). The samples were then subjected to immunoblotting for LC3B-II and β-actin. The autophagy response is shown by the LC3B-II/β-actin ratio. **c** GES-1 cells were treated with DMSO or CAG for 1 h and infected with *H. pylori* as described in (**b**). The samples were then analyzed for the protein levels of LC3B-II and β-actin by immunoblotting. **d** AGS cells were transfected with the plasmid encoding EGFP-tagged LC3 and then infected with *H. pylori* 26695 or ΔCGT as described in (**a**). The samples were then examined for autophagosomes with confocal microscopy. Cell periphery are marked with dotted-lines according to bright-field microscopy. Scale bar: 5 μm. **e** The autophagosomes observed in (**d**) were quantified by counting EGFP-LC3-positive puncta from > 25 cells in each group. **f** AGS cells transfected with the plasmid encoding EGFP-tagged LC3 were treated with DMSO or CAG, infected with *H. pylori* 26695 or ΔCGT as described in panel (**b**), and then imaged with confocal microscopy. Cell periphery are marked with dotted-lines according to bright-field microscopy. Scale bar: 5 μm. **g** Quantification of autophagosomes in (**f**). Approximately 25 cells from each group were randomly selected and counted to quantify EGFP-LC3-positive puncta. **h** GES-1 cells transfected with the plasmid of GFP-tagged LC3 were treated with DMSO or CAG, and infected with *H. pylori* 26695 or ΔCGT as described in (**b**), and then imaged by confocal microscopy. Scale bar: 10 μm. **i** The number of autophagosomes in (**h**) were quantified. Randomly ~ 25 cells from each group were examined to quantitate GFP-LC3-positive puncta. **j** Stomach cryosections of *H. pylori* 26695- and ΔCGT-infected mice were stained with anti-LC3B antibody (green) to estimate the level of induced autophagy. **k** The number of LC3-puncta in (**j**) were quantified. Randomly captured five images from each group were analyzed to quantitate LC3-positive puncta. **p < 0.01, *p < 0.05 vs. the control (n ≅ 25). In panels (**e**, **g** and **i**), data represent the mean ± SEM (standard error of the mean)

Engulfed *H. pylori* cells are sequestered inside autophagosomes [26, 36], which enhances bacterial survival [26, 36]. Residence in autophagosomes benefits the survival of intracellular *H. pylori* [26]. Therefore, to investigate the mechanistic details of bacterial-induced autophagy, we first examined the level of LC3B-II (an indicator of autophagy) in each of *H. pylori* 26695-infected and ΔCGT-infected AGS cells (Fig. 3a). The result corroborated previous findings [26, 30, 37] that *H. pylori* 26695 indeed induces a more potent autophagy response than strain ΔCGT. To identify which specific CGds contributed to this process, AGS cells pretreated with CG, CAG, or CPG were infected with ΔCGT and subjected to immunoblotting for LC3B-II (Additional file 1: Fig. S4b). Treatment with CAG induced a greater autophagy response than did CG or CPG (Additional file 1: Fig. S4b). The rescue of autophagy by CAG was examined by comparing DMSO- or CAG-pretreated AGS cells that were then infected with *H. pylori* 26695 or ΔCGT, with subsequent immunoblotting for LC3B-II (Fig. 3b). Similarly, GES-1 cells were CAG-treated, and infected with *H. pylori* for 6 h. The resulting protein levels of LC3B-II indicated that the presence of CAG promoted the autophagy response (Fig. 3c, Additional file 1: Fig. S4a). Confocal microscopy also gave a consistent result, in which AGS cells were transfected with a plasmid encoding EGFP-tagged LC3, infected with *H. pylori* 26695 or ΔCGT, and then imaged for the formation of autophagosome puncta (Fig. 3d). The formation of EGFP-LC3-labeled puncta is shown and quantitated in Fig. 3e. Strain ΔCGT induced a lesser autophagy response than did *H. pylori* 26695, indicating that CGds indeed influenced the autophagy process (Fig. 3a–e). In addition, EGFP-tagged LC3 plasmid–transfected AGS cells (Fig. 3f, g) and GES-1 cells (Fig. 3h–i) were treated with CAG, infected with *H. pylori* 26695 or ΔCGT, and imaged for the formation of autophagosome puncta. Both results revealed a significantly enhanced autophagy response in CAG-treated AGS cells in comparison with the control (Fig. 3f–i), supporting the idea that CAG contributes to *H. pylori-*induced autophagy. We then evaluated the autophagy status of the mice stomach infected with *H. pylori* 26695 or ΔCGT. Interestingly, the tissue sections of mice stomach infected with *H. pylori* 26695 displayed a higher degree of autophagy response than those infected with ΔCGT (Fig. 3j, k). The data were in agreement with the in-vitro observation that the presence of CAG stimulates more autophagy. In another perspective, this result could also be the reflection of the colonization pattern of these bacterial strains, where the more the bacterial colonization occurs, the more is the autophagy induced.

### CAG intervenes in the degradation of autophagosomes

**Fig. 4:**
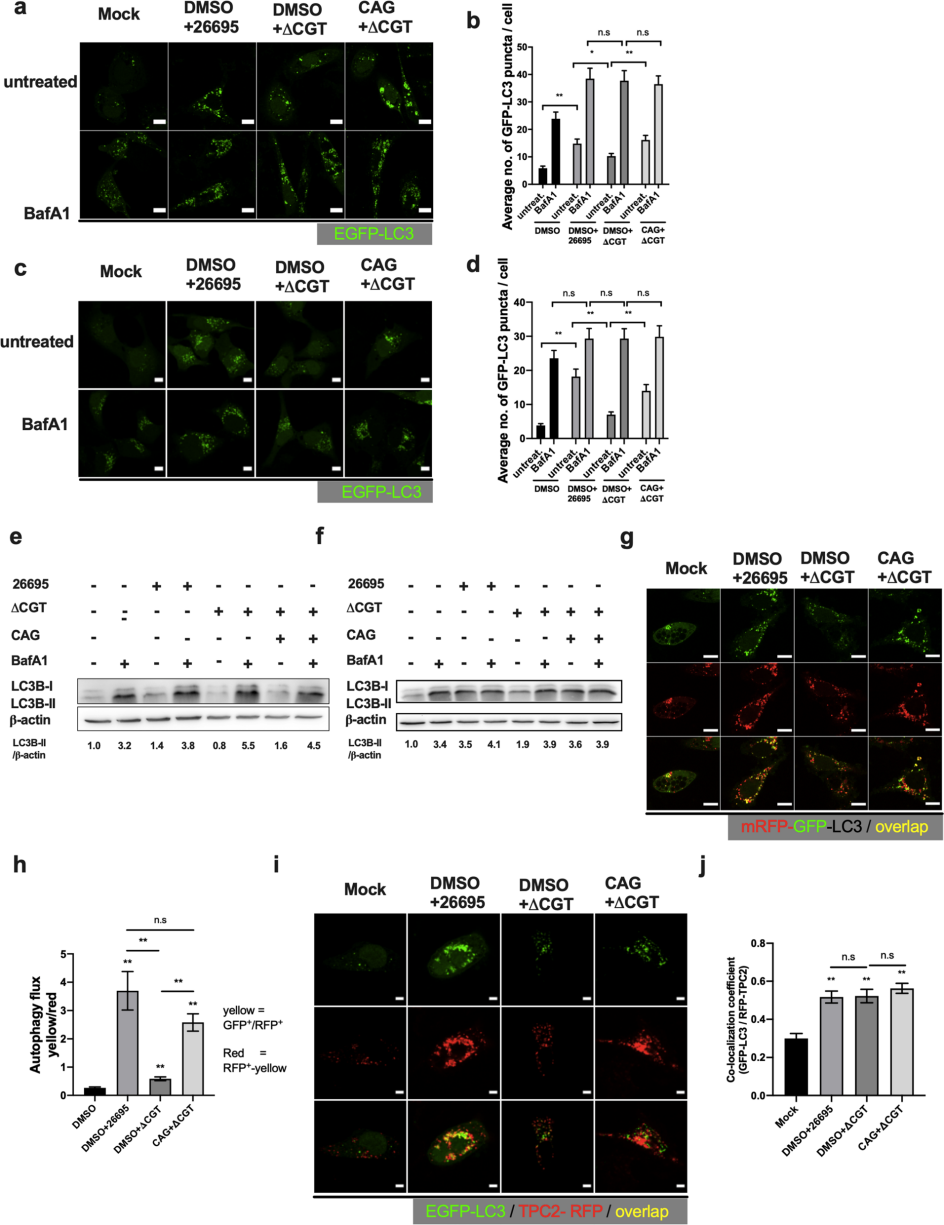
CAG impairs autophagosomal degradation. **a** AGS cells transfected with the plasmid encoding EGFP-tagged LC3 were treated with DMSO or CAG, infected with *H. pylori* 26695 or ΔCGT for 6 h, either in the absence or presence of bafilomycin A1 (BafA1, 10 nM), fixed, and imaged with confocal microscopy. Scale bar: 5 μm. **b** More than 50 cells in (**a**) that were positive for EGFP were randomly selected and counted to quantify the EGFP-positive autophagosome puncta. **c** GES-1 cells transfected with the plasmid of EGFP-tagged LC3 were treated, infected, and imaged as described in (**a**). **d** Randomly selected EGFP-positive cells were quantified for EGFP-positive autophagosome puncta. **e** AGS cells treated and infected as mentioned in (**a**) were subjected to immunoblotting for LC3B-II and β-actin. **f** GES-1 cells treated and infected as mentioned in (**a**) were examined for the protein levels of LC3B-II and β-actin by Western blotting. **g** CAG disrupts autophagy flux. AGS cells were transfected with the mRFP-GFP-LC3 tandem plasmid and then treated with DMSO or CAG, infected with *H. pylori* 26695 or ΔCGT for 6 h, and then imaged with confocal microscopy. Scale bar: 5 μm. **h** More than 50 cells from each group of (**g**) were randomly selected and counted to statistically analyze autophagy flux, shown as the yellow/red ratio. Yellow signal resulted from the puncta that are GFP^+^RFP^+^, whereas red signal originated from the RFP^+^ puncta only. **i** AGS cells were co-transfected with plasmids encoding EGFP-LC3 and TPC2-RFP, treated with CAG, and infected as indicated in (**a**). The samples were then imaged with confocal microscopy. Scale bar: 5 μm. **j** More than 20 cells that were randomly selected for quantification of EGFP and RFP fluorescence. The co-localization coefficient (signals that were positive for both TPC2-RFP and EGFP-LC3) was quantified using image J software. The data presented in panels (**b**, **d**, **h** and **j**) represent the mean ± SEM. **p < 0.01, *p < 0.05 vs. the control; n.s. (not significant)

Autophagy protects host cells against intracellular pathogens by recycling cargos via their degradation in lysosomes [15]. To counter this host defense, many intracellular pathogens have developed several strategies to intervene at various stages of autophagy [38, 39]. Although CAG increased the number of intracellular *H. pylori* (Fig. 2d, f), it remained unclear whether and how CAG regulates the autophagy response to protect the intracellular bacteria. Owing to the dynamic nature of the autophagy process, the greater numbers of EGFP-LC3 puncta or the elevated protein levels of LC3B-II could be indicative of enhanced autophagy, a blockade of fusion between autophagosomes with lysosomes, or defective degradation of autophagosome cargos in lysosomes. Bafilomycin A1 (BafA1) is a potent inhibitor of vacuolar ATPase that compromises lysosomal acidification and thus prevents autophagosome maturation. Therefore, BafA1 was used to block lysosomal degradation and cause accumulation of autophagosomes. As described above, AGS cells (Fig. 4a, b) and GES-1 cells (Fig. 4c, d) transfected with the EGFP-tagged LC3 plasmid were treated with CAG, infected with *H. pylori* 26695 or ΔCGT either in the absence or presence of BafA1 for 6 h, and subjected to imaging of the accumulation of EGFP-LC3 positive puncta. A greater number of autophagosomes were found in both *H. pylori* 26695-infected cells and CAG-pretreated ΔCGT-infected cells in the absence of BafA1 (Fig. 4a–d). In the presence of BafA1, no significant difference was observed in the number of autophagosomes in different AGS cells that were infected under different conditions (Fig. 4a–d). These results suggested that CAG (as in the infection of *H. pylori* 26695 and CAG-supplemented ΔCGT) interfered with the autophagy flux, thereby caused accumulation of autophagosomes (Fig. 4a–d). This result was also verified in AGS (Fig. 4e) and GES-1 cells (Fig. 4f) by immunoblotting for LC3B-II, which showed that the CAG-treated groups displayed a higher intensity of LC3B-II than those groups without CAG (e.g., strain ΔCGT) (Fig. 4e, f).

We next examined autophagy flux, consisting of sequential events through which engulfed cargos are ultimately dispensed to lysosomes for degradation. Autophagy flux can be measured based on the extent of lysosomal clearance of autophagosomes, which can be quantified by confocal imaging of monomeric RFP (mRFP)-GFP-tagged LC3. In accordance with this method, the green fluorescence of GFP, rather than the red signal of RFP, is quenched in acidic autolysosomes; non-acidic autophagosomes are thus yellow owing to the overlap of the GFP and RFP signals. Autophagy flux can be thus indexed by the yellow/red ratio. AGS cells were transfected with the mRFP-GFP-tagged LC3 plasmid, treated with CAG, infected with *H. pylori* 26695 or ΔCGT, and then monitored for both GFP and RFP fluorescence. Based on the yellow/red ratio, the autophagy flux was significantly perturbed in both *H. pylori* 26695-infected AGS cells and the CAG-pretreated ΔCGT-infected cells (Fig. 4g, h). By contrast, both mock- and ΔCGT-infected cells had a lower ratio, indicating a lack of perturbation of the autophagy flux.

Furthermore, because autophagosome fusion with lysosomes is a crucial checkpoint prior to lysosomal clearance of autophagosomes, we next questioned if and how it is affected by CAG. AGS cells were co-transfected with both plasmids encoding EGFP-tagged LC3 and RFP-tagged TPC2 (to label autophagosomes and lysosomes, respectively), treated with CAG, and infected with *H. pylori* 26695 or ΔCGT. Confocal microscopy was used to examine the co-localization of EGFP-labeled autophagosomes and RFP-labeled lysosomes, which revealed that autophagosome-lysosome fusion increased in each of the infected cells (i.e., *H. pylori* 26695 or ΔCGT) compared with the mock-infected cells (Fig. 4i, j); there was no significant difference among any of the infected groups. Therefore, CAG did not affect the fusion of autophagosomes with lysosomes. Taken together, we concluded that CAG interferes with the autophagy flux (Fig. 4g, h), especially with the degradation of autophagosome.

### CGAT is mainly distributed in endocytic compartments

**Fig. 5:**
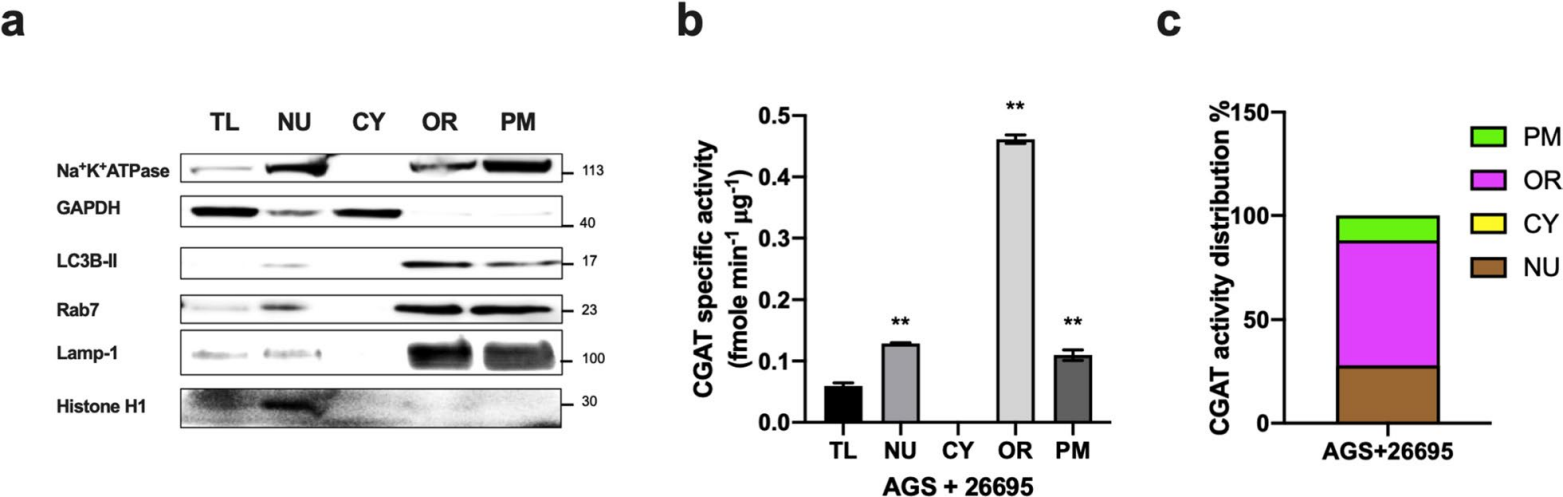
Subcellular fractionation analysis of *H. pylori-*infected AGS cells. AGS cells were infected with *H. pylori* 26695 for 6 h and lysed to generate total lysates (TL) that were subjected to subcellular fractionation using the Minute™ Plasma Membrane Extraction and Subcellular Fractionation kit. The following fractions were analyzed: nucleus (NU), cytosol (CY), organelles (OR), and plasma membrane (PM). **a** After protein quantification, TL and all fractions were examined by immunoblotting with the corresponding antibodies: anti-histone H3 (nucleus), anti-GAPDH (cytosol), anti-Rab7 and anti-LC3B (specific to late endosomes and autophagosomes, respectively), and anti-Na^+^K^+^ ATPase (plasma membrane). **b** CGAT activity of the fractions. **c** The distribution of CGAT activity in the NU, CY, OR, and PM. Total CGAT activity in each fraction was calculated by multiplying the specific activity (shown in **b**) by the amount of protein in each fraction. The plot shows the percentage of CGAT activity in each fraction. The data represent the mean ± SEM. **p < 0.01, *p < 0.05 vs. the control (n = 3)

In endosomal trafficking processes, internalized vesicles undergo homotypic fusion to form early endosomes that are then trafficked through late endosomes to lysosomes for degradation of cargos. Endosome maturation coincides with acidification, i.e., from pH 6.5 to 4.5 [40, 41]. We previously identified the acyltransferase CGAT, characterized its features and functions, and reported that its activity is optimal at pH 4.5 [21]. Because CGAT expression in host cells occurs via either bacterial internalization or the delivery of outer-membrane vesicles, we investigated whether CGAT function changes as a result of the endosomal trafficking processes. We first partitioned the lysates of *H. pylori* 26695-infected AGS cells into four subcellular fractions: plasma membrane, nucleus, cytoplasm, and organelles. Each of the fractions was examined by immunoblotting with the corresponding antibodies, i.e., anti-Na^+^K^+^ ATPase (a marker for plasma membrane), anti-Rab7 (organelles), anti-GAPDH (cytosol), anti-LC3B (autophagosomes), anti-Lamp-1 (lysosomes) and anti-histone H1 (nucleus), as shown in Fig. 5a. These fractions were assayed for CGAT activity at the same time (see “Materials and methods”). As shown in Fig. 5b, CGAT activity resided primarily in the organelle fraction that contained autophagosomes, late endosomes, and lysosomes. Figure 5c shows the percentage of CGAT activity distributed in different subcellular compartments. CGAT was enriched in endocytic organelles after the enzyme was internalized. The acidic environment apparently enhanced CGAT activity, resulting in the increased production of CAG.

### CAG reduces the lysosomal biogenesis

**Fig. 6:**
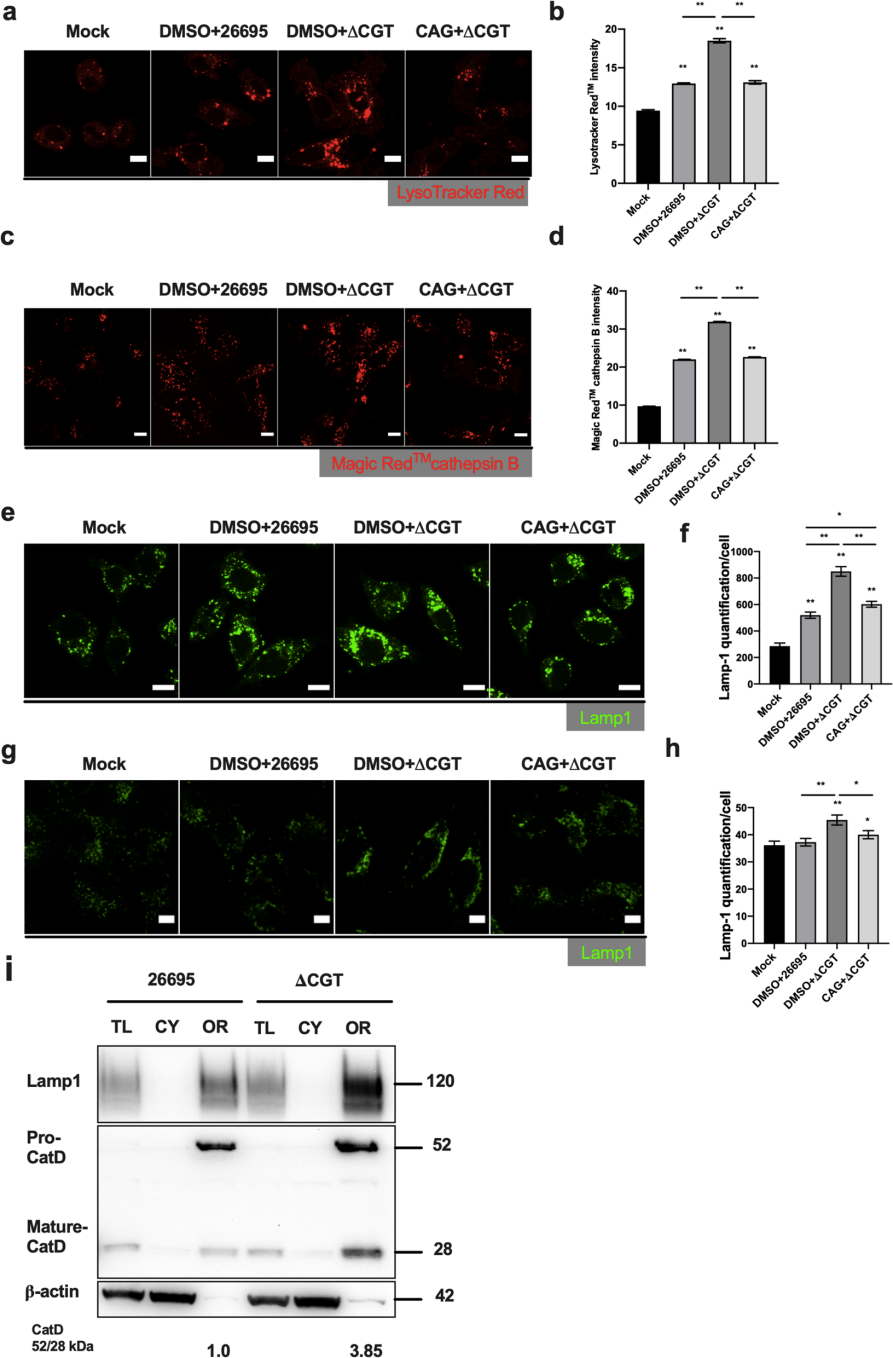
Lack of CAG increases lysosomal enzyme activity and causes accumulation of lysosomes. **a** AGS cells were pretreated with CAG, infected with *H. pylori* 26695 or ΔCGT as described in Fig. 3b, and then stained with LysoTracker™ Red DND-99 (red). Scale bar: 10 μm. **b** More than 100 cells from each group of (**a**) were counted. **c** Samples were prepared as described in Fig. 3b, followed by staining with cathepsin B substrate Magic Red™ (red). Scale bar: 10 μm. **d** More than 100 cells from each group in (**c**) were counted. **e** AGS cells treated with CAG, infected with *H. pylori* 26695 or ΔCGT as described in Fig. 3b were immunostained for Lamp-1 (green). **f** More than 100 cells from each group were counted. **g** GES-1 cells were treated with CAG, infected with *H. pylori* 26695 or ΔCGT as described in Fig. 3b, and then immunostained for Lamp-1 (green). **h** More than 50 cells from each group were counted. In panels (**b**, **d**, **f**, and **h**), the data represent the mean ± SEM. **p < 0.01, *p < 0.05 vs. the control (n ≥ 50). **i** Subcellular fractions of *H. pylori* 26695- and ΔCGT-infected AGS cells were analyzed for the levels of Lamp-1, cathepsin D, and β-actin. Thirty microgram of protein from each fraction were loaded for the analysis, according to the BCA method of protein quantification

Lysosomes contain a variety of hydrolytic enzymes to digest various biomolecules (including those of engulfed pathogens) and maintain cellular homeostasis by recycling damaged organelles via autophagy. To understand how lysosomes possibly manage *H. pylori* infection, we utilized LysoTracker™ Red (a cell-permeable acidotropic probe) to selectively label lysosomes, which have a low internal pH. More lysosomes were present in ΔCGT-infected cells than in *H. pylori* 26695-infected cells (Additional file 1: Fig. S4c, d), consistent with a previous report [30]. Prior treatment of AGS cells with CAG, i.e., before infection with ΔCGT, resulted in less-intense LysoTracker™ Red staining as compared with the same infection but without CAG (Fig. 6a, b). We then investigated if *H. pylori* infection could alter lysosomal enzyme activities. Cathepsin B is a representative lysosomal protease that is active at low pH and contributes to bacterial clearance [42]. Cathepsin B activity was measured with Magic Red™ Cathepsin B substrate after AGS cells were treated with CAG and infected with *H. pylori* 26695 or ΔCGT. Cathepsin B activity was lower in *H. pylori* 26695-infected AGS cells than in ΔCGT-infected cells (Additional file 1: Fig. S4e, f). The addition of CAG to ΔCGT-infected cells reduced cathepsin B activity (Fig. 6c, d). Next, the increased LysoTracker™ Red staining and cathepsin B activity are likely due to a result of either elevated lysosomal biogenesis or alteration in the lysosomal pH. We examined the pH and calcium level of lysosomes by using LysoSensor™ Yellow/Blue DND-160 [26] and Oregon Green™ 488 BAPTA-5N/Dextran- Alexa Fluor 568 [25], respectively. The results indicated no significant change in lysosomal pH (Additional file 1: Fig. S5a) and calcium level (Additional file 1: Fig. S5b) in *H. pylori* 26695- and ΔCGT-infected GES-1 cells, where BafA1- and ConA-treated GES-1 cells were utilized as the controls. Moreover, CAG-treated, *H. pylori*-infected AGS cells (Fig. 6e, f), and GES-1 cells (Fig. 6g, h) were immunostained for Lamp-1 and imaged by confocal microscopy. The quantification results clearly showed elevated Lamp-1 signals in ΔCGT-infected cells (Fig. 6f, h). We additionally analyzed the subcellular fractions of AGS cells infected with *H. pylori* 26695 and ΔCGT for the protein levels of Lamp1, pro- and mature-forms of cathepsin D, and β-actin. As shown in Fig. 6i, both Lamp-1 expression, and pro- to mature-cathepsin ratio were significantly increased in ΔCGT-infected AGS cells as compared to the other counterpart [30]. These results indicated that ΔCGT bacterial infection indeed elicits a higher level of Lamp-1 biogenesis. Bacterial clearance is more efficient when cells have a greater number of lysosomes with higher enzyme activities. Consistent with this fact, we observed that greater autophagy flux coincided with a greater number of lysosomes and higher cathepsin B activity. These results implied that ΔCGT was degraded more effectively by lysosomes. In other words, CAG was able to reduce lysosomal clearance of autophagosomes.

### Autophago-lysosomes harbor intracellular *H. pylori*

**Fig. 7:**
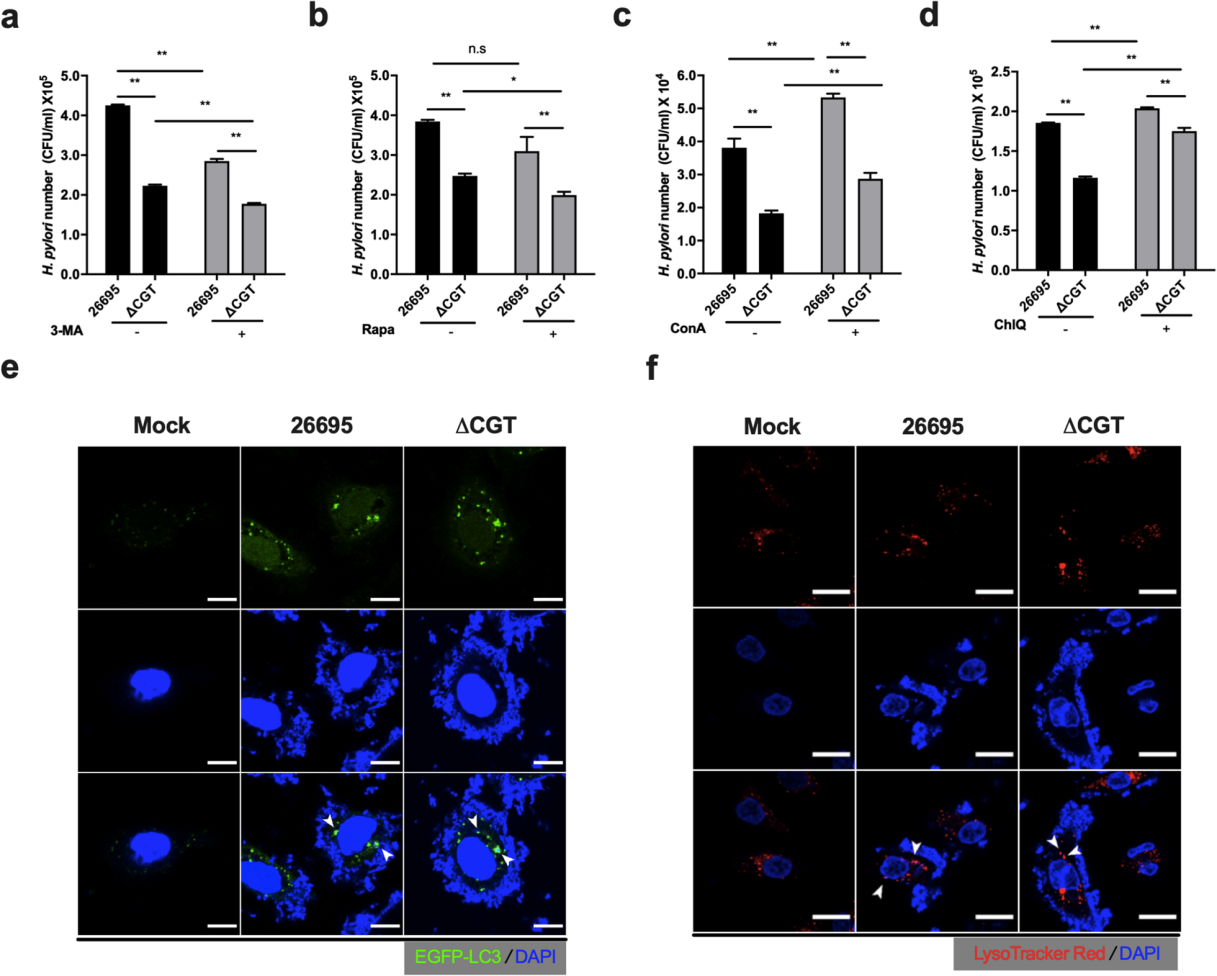
Defective autophagosomes harbor intracellular *H. pylori*. AGS cells were left untreated or pretreated with pharmacological drugs: **a** 3-methyl adenine (3-MA, 2 mM), **b** rapamycin (Rapa, 100 nM), **c** concanamycin A (ConA, 50 nM), or **d** chloroquine (ChlQ, 10 μM). The samples were then infected with *H. pylori* 26695 or ΔCGT and treated with gentamycin for 1 h. The samples were then lysed and plated for colony counting (CFU/ml). Data represent the mean ± SEM. **p < 0.01, *p < 0.05 vs. the control (n = 3). **e** AGS cells transfected with the plasmid encoding EGFP-tagged LC3 were treated and infected as indicated in Fig. 3a and then stained with DAPI (blue) to label both host-cell nuclei and bacteria. Scale bar: 5 μm. **f** After AGS cells were infected with *H. pylori* 26695 or ΔCGT as indicated in Fig. 3a, they were stained with LysoTracker™ Red DND-99 (red) and labeled with DAPI (blue) to label both host-cell nuclei and bacteria. Scale bar: 10 μm

To investigate how internalized *H. pylori* survives in non-degradative autophagosomes [26, 36], 3-methyl adenine and rapamycin were utilized to inhibit and induce autophagosome formation, respectively. Rapamycin not only induces autophagosome formation but also facilitates efficient autophagy turnover [26]. In line with the report of Zhang and coworkers [26], both inhibition (Fig. 7a) and induction (Fig. 7b) of autophagosome formation resulted in the timely clearance of intracellular *H. pylori*, regardless of the bacterial strain used. Furthermore, three pharmacological inhibitors were used to disrupt lysosomal degradative function, including BafA1, concanamycin A (potently inhibits vacuolar ATPase), and chloroquine (impairs autophagosome fusion with lysosomes and alters lysosomal pH, i.e., because it is a lysosomotropic weak base, to inhibit enzyme activities). These inhibitors significantly increased the number of intracellular *H. pylori* (Fig. 7c, d and Additional file 1: Fig. S5c) in both *H. pylori* 26695-infected and ΔCGT-infected AGS cells. Because the number of intracellular *H. pylori* 26695 was higher as compared with ΔCGT, CAG appeared to disrupt the progression of autophagosomes that then served as a shelter for bacteria. Therefore, the lack of autophagosomes resulted in lower survival, as shown by the inhibition or induction of autophagosome formation (Fig. 7a, b, respectively). In contrast, perturbation of autophagosome maturation enhanced survival (Additional file 1: Fig. S5c, Fig. 7c, d). AGS cells were also transfected with the EGFP-tagged LC3 plasmid and infected with *H. pylori*, as mentioned above, and then labeled with DAPI to stain the DNA of both *H. pylori* and AGS cells*.* Intracellular *H. pylori* co-localized with EGFP-tagged autophagosomes (Fig. 7e). To evaluate whether the bacteria were also present in lysosomes, the DNA of *H. pylori* and AGS cells were labeled with DAPI and lysosomes with LysoTracker™ Red. Confocal imaging revealed the co-localization of intracellular *H. pylori* with lysosomes in the infected AGS cells (Fig. 7f). Taken together, these results indicated that *H. pylori* induces autophagy, but the accompanying formation of dysfunctional autophago-lysosomes cannot afford their efficient degradation and thus those autophago-lysosomes become a shelter for *H. pylori*.

## Discussion

CGAT is active between pH 3 and 9, with optimal activity at pH 4.5 [21]. In particular, the activity at pH 4.5 is twice that at pH 7.0 [21]. Our results indicate that the majority of CGAT activity was in the organelle-containing fraction upon bacterial internalization. The low pH of endocytic compartments, such as autophagosomes (pH 5.0) and lysosomes (pH 4.5), reveals an effective strategy of using CGAT*.* Once the bacteria are internalized and sequestered via autophagy, CGAT activity gradually increases to produce more CAG in each acidic environment.

The *H. pylori* CGAT is not just an enzyme required for the formation of CAG. It has multifaceted functional roles. Previous studies indicated that CGAT is essential for bacterial adhesion, and inhibiting its activity effectively abolishes the adherence of *H. pylori*. In several multidrug-resistant strains of *H. pylori*, the CAG level was 10- to 150-times higher than that of *H. pylori* 26695. This observation implies that CGAT may be abundant in clinical isolates of *H. pylori*. Moreover, CGAT is found in outer-membrane vesicles [21]. It can be thus delivered to the host cells without direct bacterial adhesion and invasion. Consequently, the host autophagy defense can be compromised prior to bacterial adhesion as long as there is release and fusion of bacterial outer-membrane vesicles with the host-cell plasma membrane.

In fact, there are no thorough studies regarding the influence of membrane lipids on the structure and function of autophagosomes. Owing to the technical difficulties associated with isolating homogeneous subcellular and autophagic membranes, the exact lipid composition of these membranes is unknown. The interplay between membrane lipids and proteins is crucial for bacterial internalization. *Listeria monocytogenes*, for instance, secretes two phospholipases that interfere in the efficient generation of phosphatidylinositol 3-phosphate, leading to stalling of pre-autophagosome structures and decreased autophagy flux [43]. Our results do not only demonstrate the effect of CAG on the negative regulation of the host autophagy-mediated degradation, but also underscore the fact that CGAT activity is maximal in an acidic environment such as that of the autophagosome and lysosome. The elevated CGAT activity clearly explains how intracellular bacteria leverage their lipid and lipid-biosynthesis enzymes to regulate degradation of endosomes in host cells. The findings indicate an important connection between bacterial CGAT and the function of autophagosomes and lysosomes in host cells.

**Fig. 8:**
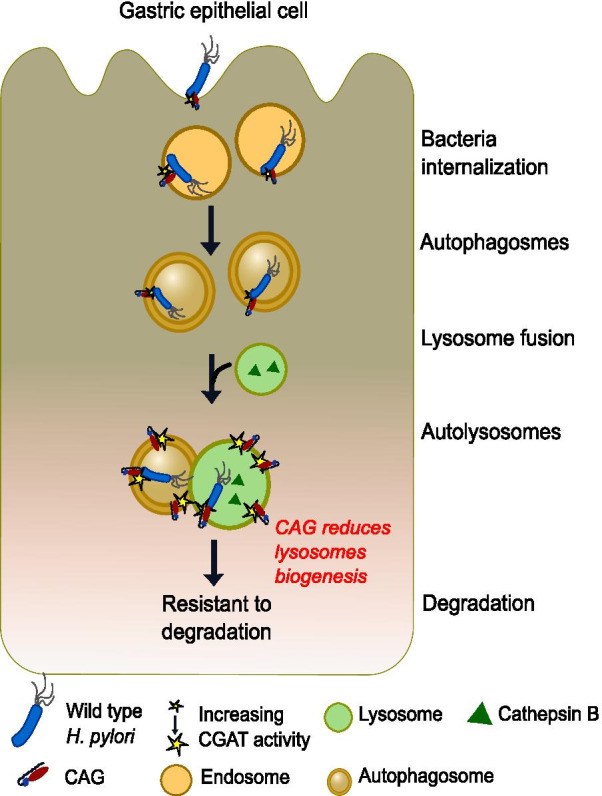
Schematic presentation explains how *Helicobacter pylori* hijacks host-cell autophagy to favor the intracellular survival. *H. pylori* is a cholesterol auxotroph and converts cholesterol to cholesteryl 6ʹ-*O*-acyl-α-d-glucoside (CAG) by the enzymes of cholesteryl glycosyltransferase and the acyltransferase (CGAT). Our results support the idea that CGAT is mainly distributed in the subcellular compartment consisting of autophagosomes and late endosomes, in which the acidic environment is necessary for maximal enzyme activities. The elevated level of CAG can facilitate bacterial internalization, interfere with the autophagy flux, and cause reduced lysosomal biogenesis

In this study, CAG of *H. pylori* was identified as a compound that promotes bacterial internalization, yet the same compound averted lysosomal clearance of autophagosomes, thereby favoring bacterial survival. This work demonstrates an effective strategy by which *H. pylori* manipulates the host autophagy for its own benefit (Fig. 8). Although functions for CGds were hypothesized previously [30, 44] with respect to their participation in the survival of intracellular bacteria in macrophages, the mechanistic details for any particular CG remained elusive. Our current results provide evidence to support a substantive role for CAG and the related enzyme CGAT in preventing lysosomal clearance of autophagosomes.

*H. pylori* employs cholesterol-rich lipid rafts for the delivery of two virulence factors (including cytotoxin-associated gene A [22] and vacuolating cytotoxin A (abbreviated as CagA and VacA, respectively) [45], oligomerization of VacA [36, 46], and escape from the host’s IFN-γ inflammatory response [47, 48]. The CAG-induced clustering of lipid rafts [22] was previously attributed to enhance bacterial adhesion [21, 23, 49]. We showed that the depletion of cholesterol-rich lipid rafts significantly decreased the internalization of both *H. pylori* and CAG. Because the addition of CAG to ΔCGT-infected AGS cells increased the incidence of bacterial internalization, CAG likely acts synergistically with cholesterol at the bacterial entry site of host cells, which gathers lipid raft-associated components into membrane ruffles for efficient engulfment. In agreement with this explanation, Lafong et al. reported that lipid rafts help bacteria form membrane invaginations for effective internalization [9], as demonstrated for *M. tuberculosis* [50] and pathogenic *E. coli* [12].

CGds constitute a substantial proportion of plasma membrane lipids in *H. pylori* [51] and are indispensable for membrane integrity. Lack of CGds (e.g., strain ΔCGT) results in an abnormal lipopolysaccharide profile [34]. In this study, we additionally observed that the absence of CAG was associated with increased lysosomal enzyme activities. This effect of a lack of CAG likely explains why CAG-producing *H. pylori* are resistant to lysosome-mediated elimination. A recent study indicated that epithelial cells, upon chronic exposure to VacA, have decreased cathepsin D activity and inefficient bacterial eradication, although autophagosome maturation was not perturbed [52]. Similarly, we observed that the presence of CAG decreased cathepsin B activity yet did not affect the fusion of autophagosomes with lysosomes.

Lipid rafts are often associated with bacterial internalization, which determines whether intracellular bacteria are delivered to lysosomes [53]. For example, the Gram-negative bacterium *Brucella abortus* is able to resist degradation when it is contained in lipid raft-enriched vacuoles [54]. The parasite *Leishmania donovani* uses lipophosphoglycans to alter the organization of lipid rafts on the phagosomal membrane of macrophages, which disrupts the recruitment of host-cell lysosomal-associated membrane protein 1 (to maintain lysosomal integrity, pH and catabolism) [55], thus providing a survival advantage [56]. Likewise, we noticed fewer lysosomes in AGS cells after infection with CAG-producing *H. pylori*. Because *H. pylori* infection alters the CAG composition of the host-cell plasma membrane [21, 22], it would be intriguing to investigate whether a similar change occurs in the lysosome membrane, and if any such change occurs, then what is the connection with the reduction in lysosomal enzyme activities. *H. pylori* VacA inhibits the lysosomal calcium channel protein, TRPML1, to disrupt endo-lysosomal trafficking, resulting in defective lysosomes that could be leveraged by *H. pylori* as an intracellular reservoir to favor bacterial recurrence upon withdrawal of antibiotics [25]. As mentioned above, VacA relies on host-cell lipid rafts for its activation to cause disruption in the endo-lysosomal compartments without affecting autophagosome maturation, which is reminiscent of our CAG studies. In particular, VacA is a pore-forming toxin that associates with endosomal and mitochondrial membranes [57]. It thus would be intriguing to understand if there is a correlation between CAG-mediated formation of lipid rafts and VacA-associated virulence—particularly any possible synergistic relationship to regulate lysosomal function.

## Conclusions

Our results support the idea that the acyltransferase is mainly distributed in the subcellular compartment consisting of autophagosomes, late endosomes, and lysosomes, in which the acidic environment is beneficial for the maximal acyltransferase activity. The resulting elevated level of CAG can facilitate bacterial internalization, interfere with the autophagy flux, and causes reduced lysosomal biogenesis.

## Supplementary Information

**Additional file 1. Fig. S1.** Chemical structures of CG-MAN, CAG-MAN, and CPG-MAN. **Fig. S2.** Inhibition of cholesterol biosynthesis abolishes the internalization of CAG-MAN and CPG-MAN by AGS cells. **Fig. S3.** Co-localization of puncta of CAG-MAN and CPG-MAN with intracellular *H. pylori* 26695. **Fig. S4.** The autophagy response induced by ΔCGT in the presence of CG, CAG, or CPG. **Fig. S5.** Lysosomal pH and calcium levels remained unaffected by the infection with *H. pylori* 26695 or ΔCGT and the treatment with bafilomycin A1 increased the number of intracellular *H. pylori*.

## Data Availability

The datasets used and analyzed during the current study are available from the corresponding author on reasonable request.

## Abbreviations

CGds: Cholesterol α-d-glucoside derivates
CAG: Cholesteryl 6′-*O*-acyl-α-d-glucopyranoside
CGAT: Cholesteryl α-d-glucopyranoside 6′-acyltransferase
MAN: 4-*N*-Methylamino-1,8-napthalimidopropyne
