# Supplementary material for: Enhanced enzymatic production of cholesteryl 6ʹ-acylglucoside impairs lysosomal degradation for the intracellular survival of Helicobacter pylori
Source: J Biomed Sci. 2021 Oct 27;28:72. doi: 10.1186/s12929-021-00768-w (PMC8549234; doi:10.1186/s12929-021-00768-w)

Supplementary Figure 1

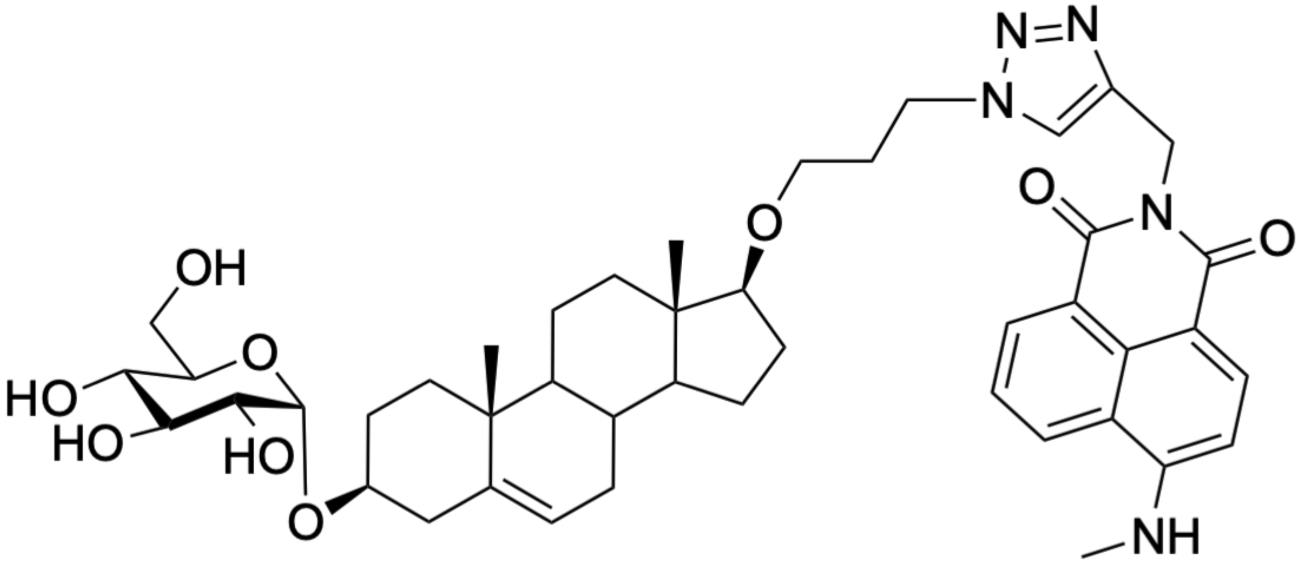

CG-MAN

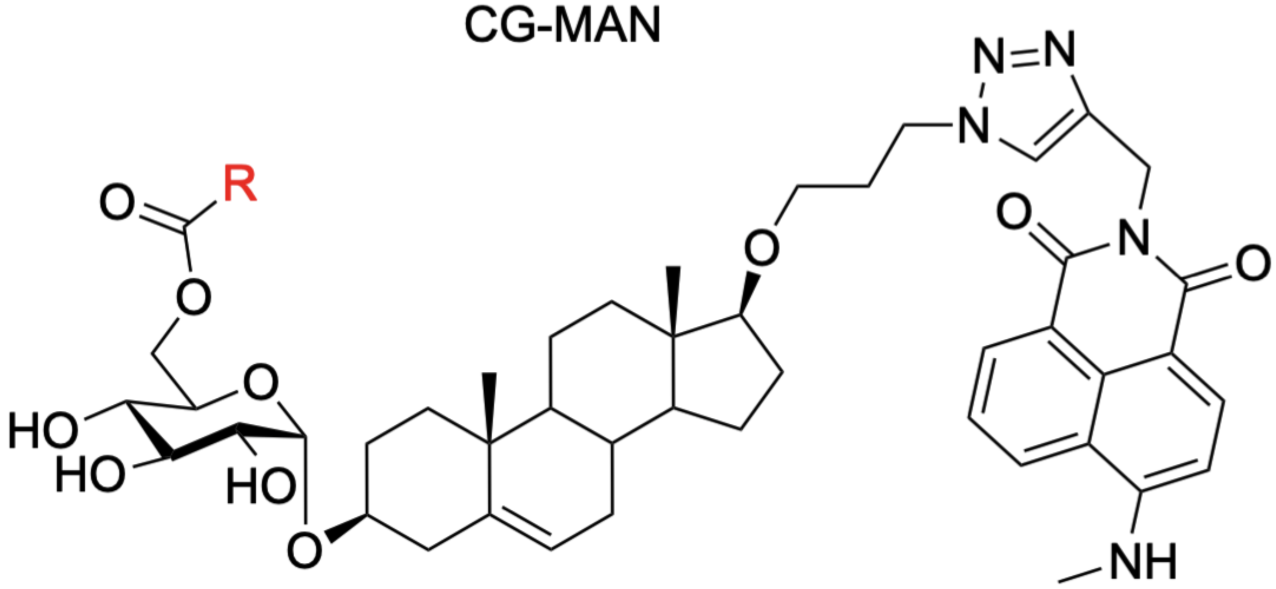

CAG-MAN

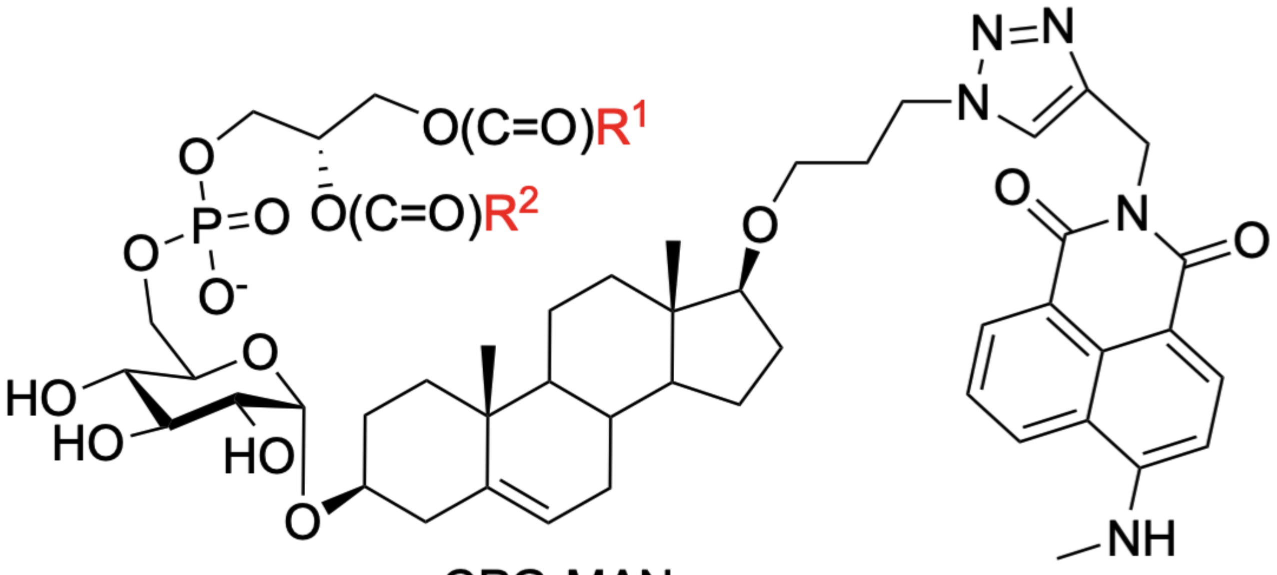

CPG-MAN

R, R1, R2 = alkyl

Supplementary Figure 2

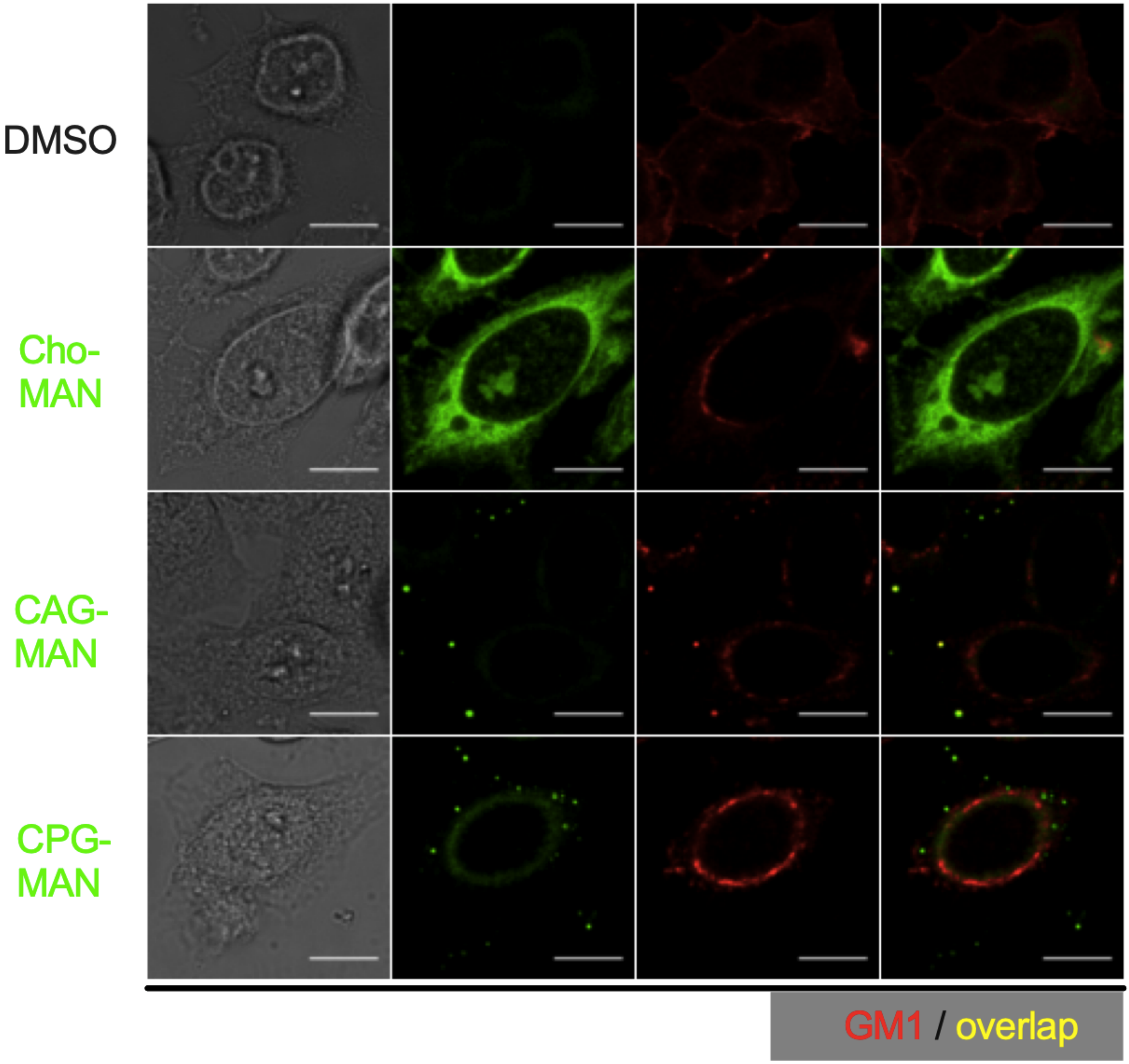

Supplementary Figure 3

a

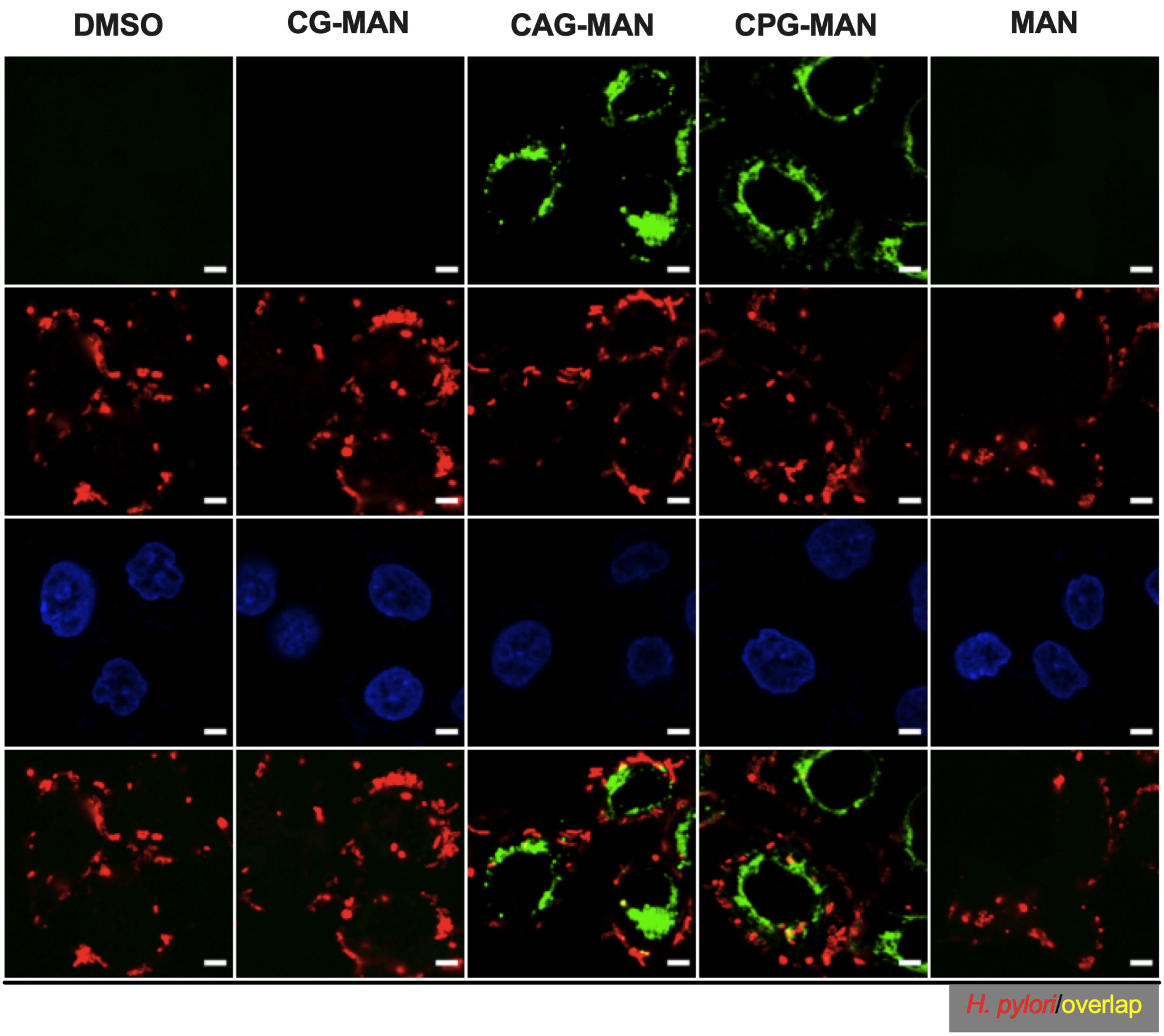

b

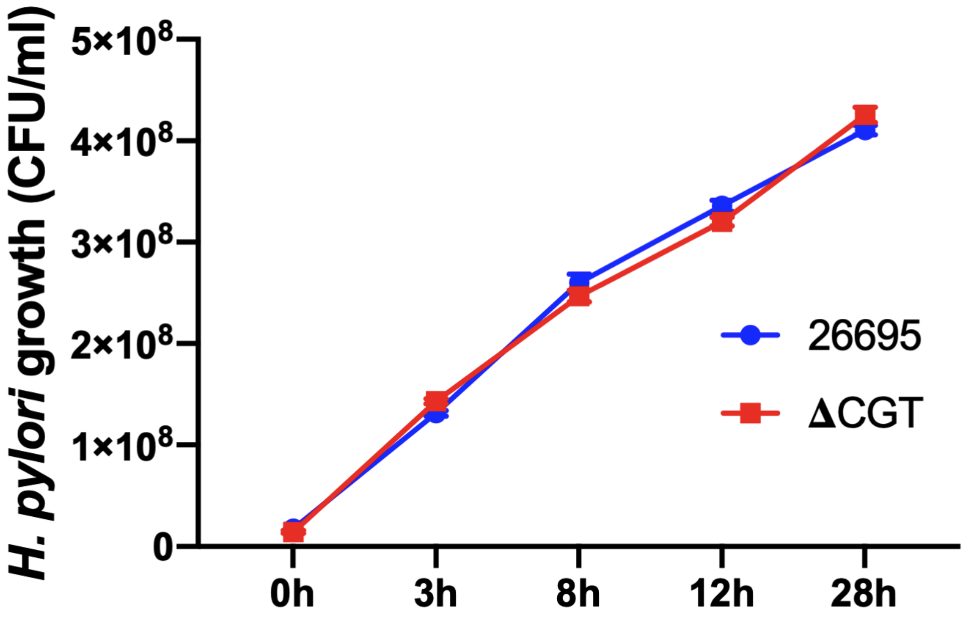

Supplementary figure. 4

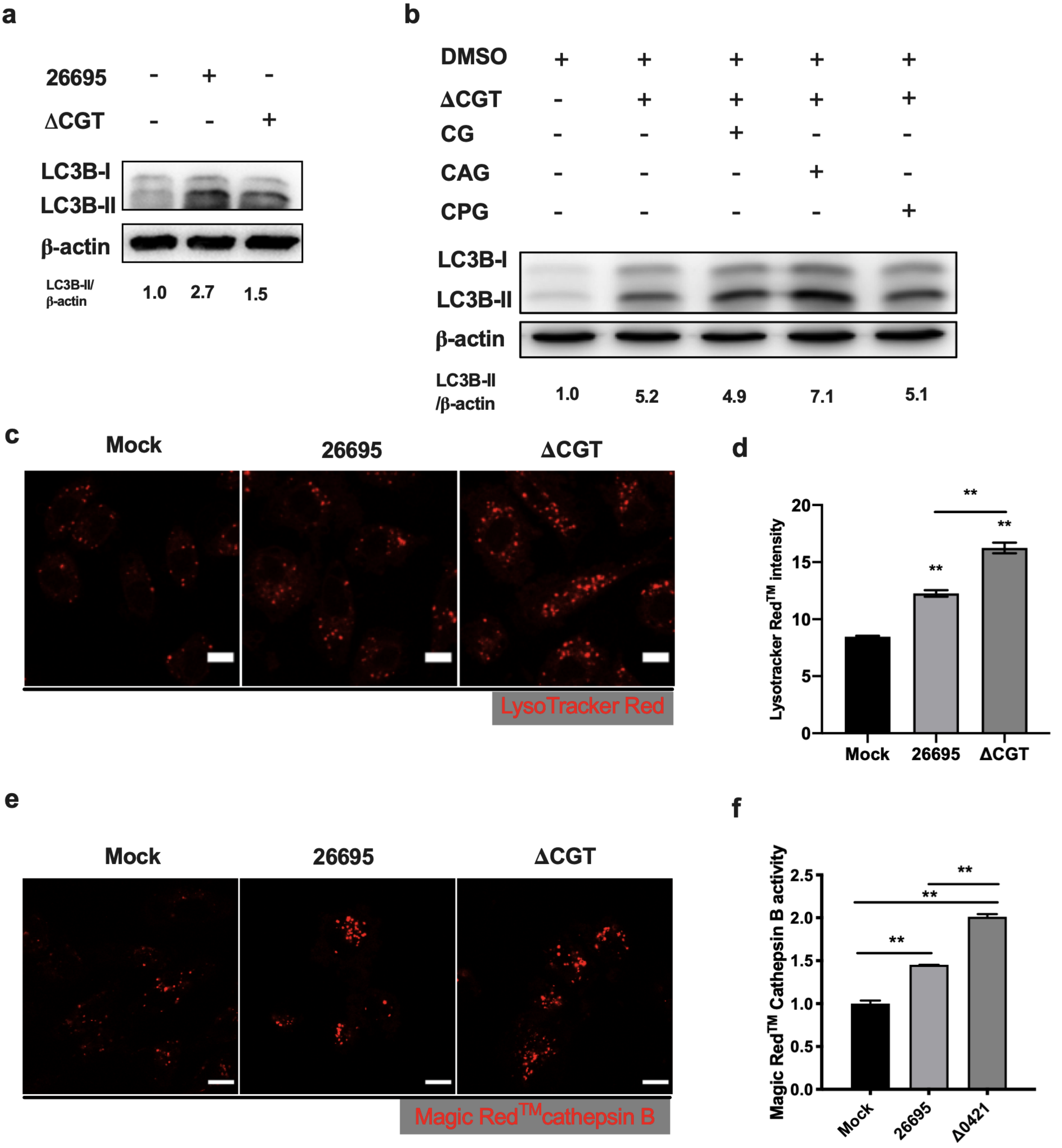

Supplementary Figure 5

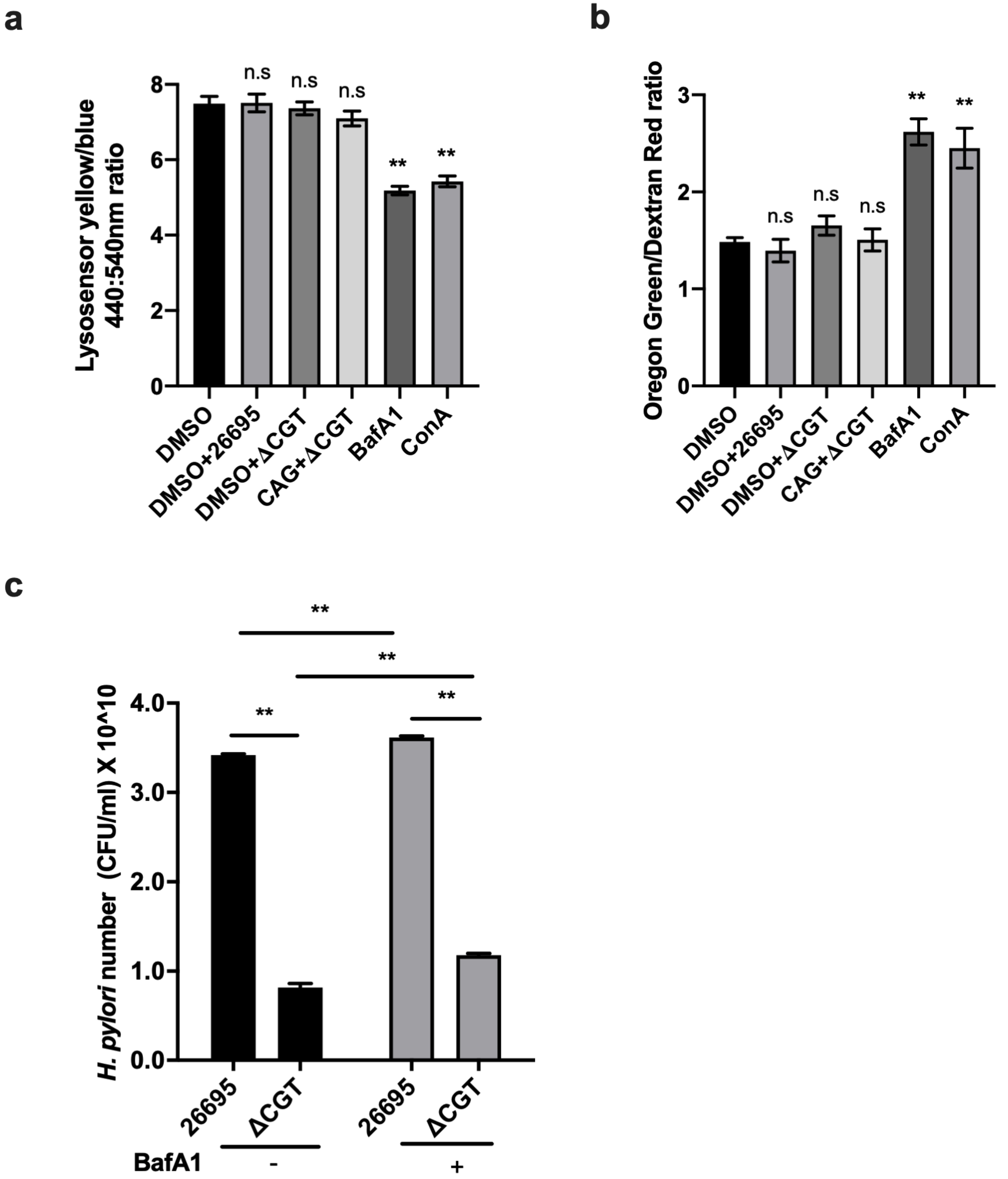

Supplement: Supplementary file 1 — Additional file 1. Fig. S1. Chemical structures of CG-MAN, CAG-MAN, and CPG-MAN. Fig. S2. Inhibition of cholesterol biosynthesis abolishes the internalization of CAG-MAN and CPG-MAN by AGS cells. Fig. S3. Co-localization of puncta of CAG-MAN and CPG-MAN with intracellular H. pylori 26695. Fig. S4. The autophagy response induced by ΔCGT in the presence of CG, CAG, or CPG. Fig. S5. Lysosomal pH and calcium levels remained unaffected by the infection with H. pylori 26695 or ΔCGT and the treatment with bafilomycin A1 increased the number of intracellular H. pylori. [file 12929_2021_768_MOESM1_ESM.pdf]
